# Trends and frontiers in disuse muscle atrophy research

**DOI:** 10.3389/fpubh.2025.1611571

**Published:** 2025-11-17

**Authors:** Shenghui Wu, Yu Miao, Jiong Mei, Shengren Xiong

**Affiliations:** 1 Department of Orthopedic Surgery, Fujian Medical University Union Hospital, Fujian, China; 2 Department of Orthopaedic Surgery, Shanghai Sixth People's Hospital Affiliated to Shanghai Jiao Tong University School of Medicine, Shanghai, China

**Keywords:** disuse muscle atrophy, bibliometric analysis, PubMed, visuliasation, R software

## Abstract

**Background:**

To investigate the progress and status of the disuse muscle atrophy (DMA) and to highlight the current hot research areas.

**Materials and methods:**

A comprehensive literature search was conducted in the PubMed database, covering the period from 2010 to 2024. The R software (version 4.2.0) was used for the bibliometric analysis.

**Results:**

A total of 689 articles related to DMA have been published, demonstrating a consistent upward trend in research output. The University of Utah was identified as the most productive institution in this field, while the United States led in the number of publications. Notable authors, including Marlou L. Dirks and Benjamin T. Wall, were recognized as the most prolific contributors, significantly advancing the literature. Key journals such as the Journal of Applied Physiology, Journal of Cachexia, Sarcopenia and Muscle, and International Journal of Molecular Sciences were highlighted for their high citation metrics and considerable influence. Hot keywords, such as “oxidative stress,” “aging,” “exercise,” “protein synthesis,” “mitochondria,” “inflammation,” “Murf1,” “soleus muscle,” “Foxo3,” “ROS,” and “glucocorticoids,” were the most frequently occurring terms. Additionally, most countries were actively engaged in extensive collaborative networks.

**Conclusion:**

This study highlighted an increasing emphasis on molecular mechanisms of DMA, such as oxidative stress, aging, protein synthesis, mitochondrial function, and inflammation. Murf1 and Foxo3 played crucial roles in the process of DMA. Both exercise and glucocorticoids were primary methods for enhancing muscle recovery. The soleus muscle was the primary focus in DMA studies. This study provided a valuable reference for guiding future research and optimizing potential intervention strategies for DMA.

## Introduction

Disuse muscle atrophy (DMA) was a condition characterized by the loss of skeletal muscle mass and strength due to periods of inactivity or reduced physical activity. This condition can arise from various circumstances, including immobilization, bed rest, or reduced physical activity due to illness or injury. The mechanisms underlying disuse-induced muscle atrophy were complex and multifaceted, involving a combination of decreased muscle protein synthesis and increased protein breakdown. During periods of inactivity, the balance between these two processes was disrupted, leading to net muscle loss.

One of the primary contributors to disuse muscle atrophy was the transformation of muscle fibers from a slow, oxidative phenotype to a fast, glycolytic one. This shift was accompanied by a decline in mitochondrial density and function, which further exacerbated muscle fatigue and weakness (1). Additionally, periods of disuse were associated with increased production of reactive oxygen species (ROS), which played a critical role in regulating redox-sensitive signaling pathways that can control both protein synthesis and degradation in skeletal muscle (2). The accumulation of ROS can lead to oxidative stress, further promoting muscle atrophy. Nutritional strategies, such as optimizing protein intake, have been proposed as potential countermeasures to mitigate disuse-induced muscle atrophy. Protein-rich diets and supplementation with amino acids can help preserve muscle mass and function by stimulating muscle protein synthesis and reducing protein breakdown (3). Furthermore, resistance exercise was recognized as a potent anabolic stimulus that can effectively counteract muscle losses during periods of disuse. However, the feasibility of performing resistance exercise during illness or injury may be limited, particularly in older adults (4). Thus, DMA was a significant concern in various clinical and non-clinical settings. Understanding the underlying mechanisms and exploring effective interventions, such as nutritional strategies and resistance exercise, were crucial for mitigating the adverse effects of muscle atrophy and promoting recovery following periods of inactivity.

Bibliometric analyses served as a powerful tool in identifying research gaps and revealing evolving trends in both clinical and basic research fields, such as DMA. By systematically analyzing large volumes of scientific literature, these analyses can highlight areas that required further investigation and provide insights into the trajectory of research over time. In the realm of muscle research, bibliometric analyses have been used to map out the landscape of quantitative ultrasound assessments of skeletal muscle, which was crucial for early detection of muscle dysfunction and sarcopenia. Such studies have identified key trends, gaps, and themes that guided future research and clinical applications, emphasizing the utility of quantitative ultrasound as a critical tool in assessing skeletal muscle mass and function (5). Similarly, the intersection of sarcopenia and microbiome research has been explored through bibliometric methods, revealing emerging research hotspots such as malnutrition, dietary fiber, and signaling pathways. This analysis underscored the nascent stage of research on the impact of the microbiome on sarcopenia and highlighted the multidisciplinary nature of this field, which can span nutrition, microbiology, geriatrics, and more (6). Furthermore, bibliometric analyses can provide a comprehensive overview of global research trends, as seen in studies of spinal muscular atrophy (SMA). These analyses have highlighted significant advancements in SMA research, including genetic and molecular mechanisms, survival motor neuron gene therapy, and muscle strength-promoting factors. Such insights were invaluable for aligning research efforts with global medical advancements and improving the lives of individuals affected by neuromuscular diseases (7). Therefore, bibliometric analyses were instrumental in mapping the research landscape, identifying gaps, and guiding future investigations in various fields, including disuse muscle atrophy. By leveraging these analyses, researchers can better understand the current state of knowledge and strategically focus their efforts on areas with the greatest potential for impact.

This study aimed to conduct a bibliometric analysis of literature related to DMA. We hypothesized that identifying systematic academic frameworks and trend analyses in the research on DMA can contribute to the advancement of this field.

## Methods

**Figure 1:**
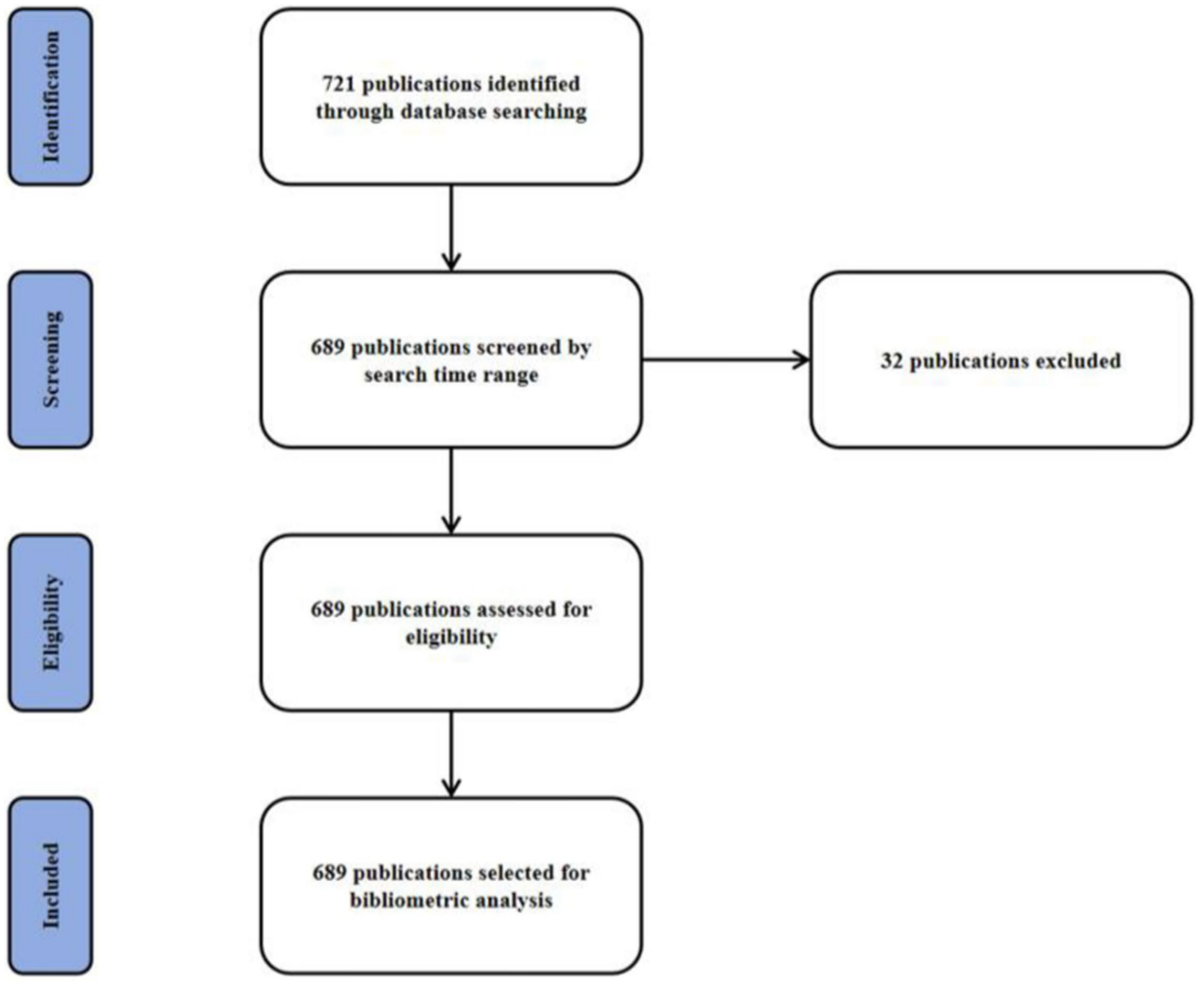
The PRISMA flow chart of bibliometric analysis of publications on disuse muscle atrophy.

In our study, we aimed to provide a comprehensive overview of the research landscape on DMA. Therefore, we did not apply specific exclusion criteria beyond the search strategy parameters (e.g., language, document type). All records retrieved that matched the search terms were included in the analysis to avoid introducing subjective bias and to ensure that the bibliometric mapping reflected the full scope of published DMA-related literature. The PRISMA flow chart was shown (Figure 1). This research utilized the R package “bibliometrix” (version 4.1.4)^1^ for comprehensive bibliometric analysis, constructing a global literature network focused on the DMA. The search strategy was ((Disuse Muscle Atrophy[topic]) OR (Muscle Disuse Atrophy[topic]) OR (Disuse Atrophy[topic]) OR (Skeletal Muscle Atrophy[topic]) OR (Muscle Wasting[topic]) OR (Muscle Deconditioning[topic])) AND (Physical Inactivity[topic]) OR (Unloading[topic]) OR (Immobilization[topic]) OR (Bed Rest[topic]) OR (Microgravity[topic])). The analysis covered journals, authors, citations, keywords, institutions, countries, and co-occurrence networks. Complete metadata were extracted from the PubMed database on March 17, 2025, spanning from January 1, 2010, to December 31, 2024, in the “PubMed export file” format and processed using R (version 4.2.0) with the Bibliometrix package. Initial data assessment was conducted using the “biblioAnalysis()” command and “summary()” function, providing insights into the temporal distribution of articles, document types and quantities, average publication year, citation metrics, authorship patterns, and the geographical distribution of corresponding authors. The analysis also identified prolific authors, highly cited manuscripts, and key publication sources. Feature mapping and collaboration networks were developed using the “metaTagExtraction” and “Biblionetwork” commands, with visualizations generated via “Networkplot.” Additional analyses, including national and institutional collaboration networks, keyword analysis, co-occurrence network synthesis, and thematic mapping, were performed using the “Biblioshiny()” command.

## Results

### Annual publication trends analysis

**Figure 2:**
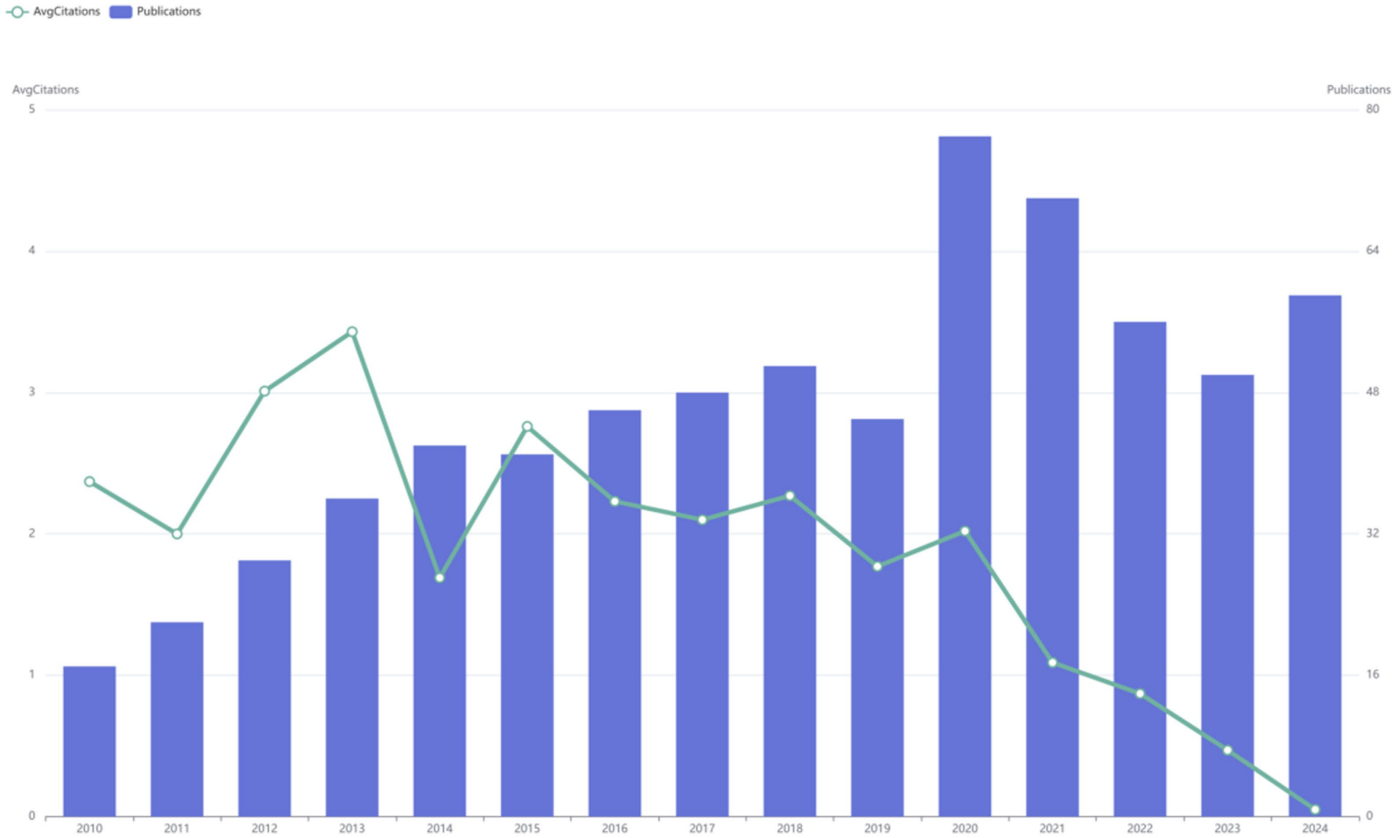
Annual publication trends and average citation counts of literature at the disuse muscle atrophy from January 2010 to December 2024.

This search yielded 689 publications from January 2010 to December 2024, with an average annual publication count of 46. As illustrated, research related to the DMA can be broadly divided into two phases. The initial phase of the study was marked by a period of continuous growth. In 2020, the number of publications reached 77, representing a 1.7-fold increase from 2019. Between 2010 and 2019, the average annual number of papers published was 38. However, after 2021, there was a sharp decline in the volume of literature. Additionally, the highest annual average citation count was recorded in 2013 at 3.43, after which it declined significantly. By 2015, the average citation count had dropped to 2.76 and continued to decrease annually thereafter (Figure 2).

### Three-field plot

**Figure 3:**
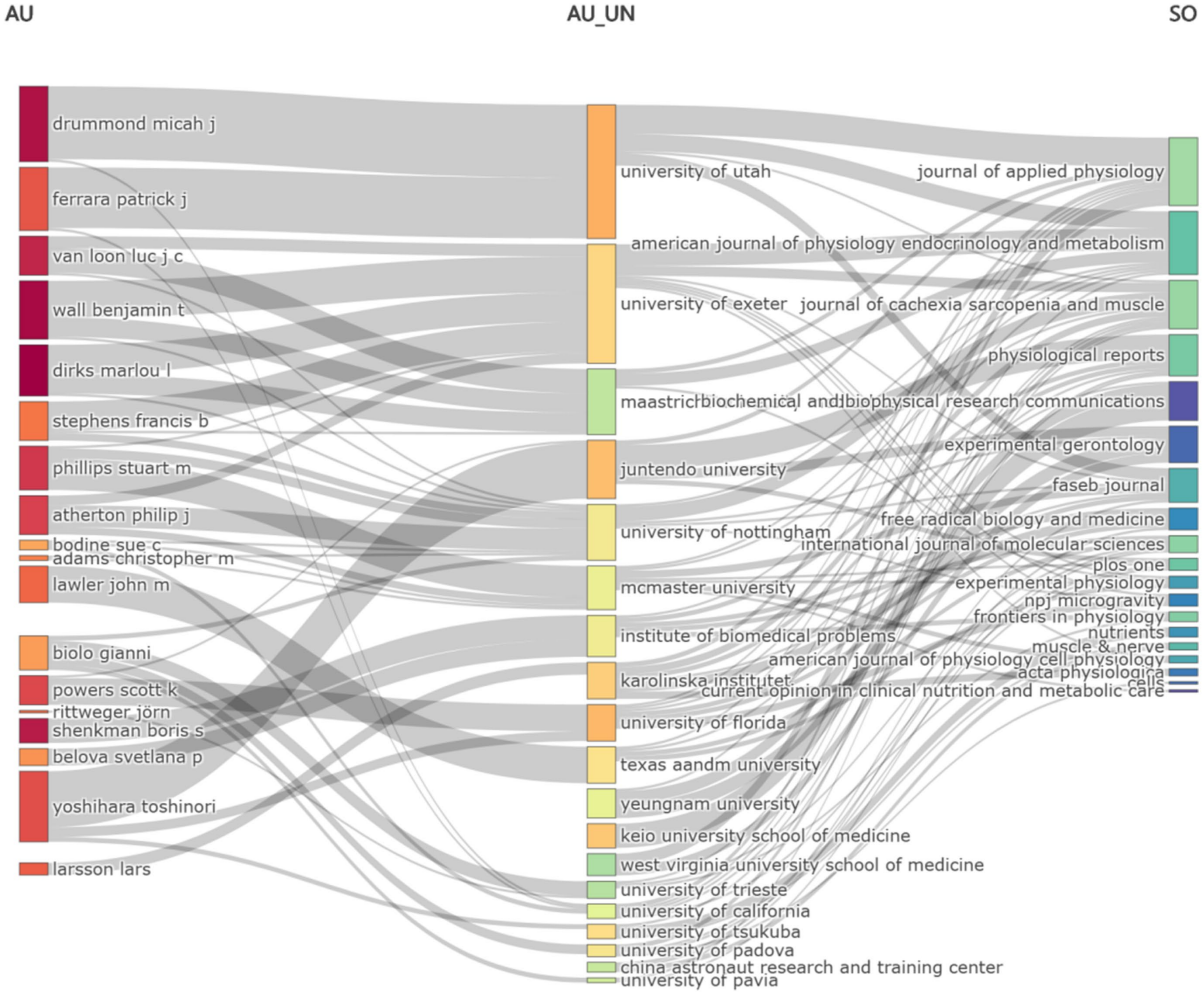
Sankey diagram of literature on the disuse muscle atrophy (Fields: AU—AU_UN—SO) from January 2010 to December 2024.

The Sankey diagram, illustrating the relationship among Author (AU), Affiliation (AU_UN), and Source (SO) in global literature related to the DMA, revealed the flow and connections between these fields (Figure 3). Notably, the highest flow from the Author category was directed toward the University of Utah, highlighting its significant role in the DMA.

### Most relevant sources and sources’ production over time

**Figure 4:**
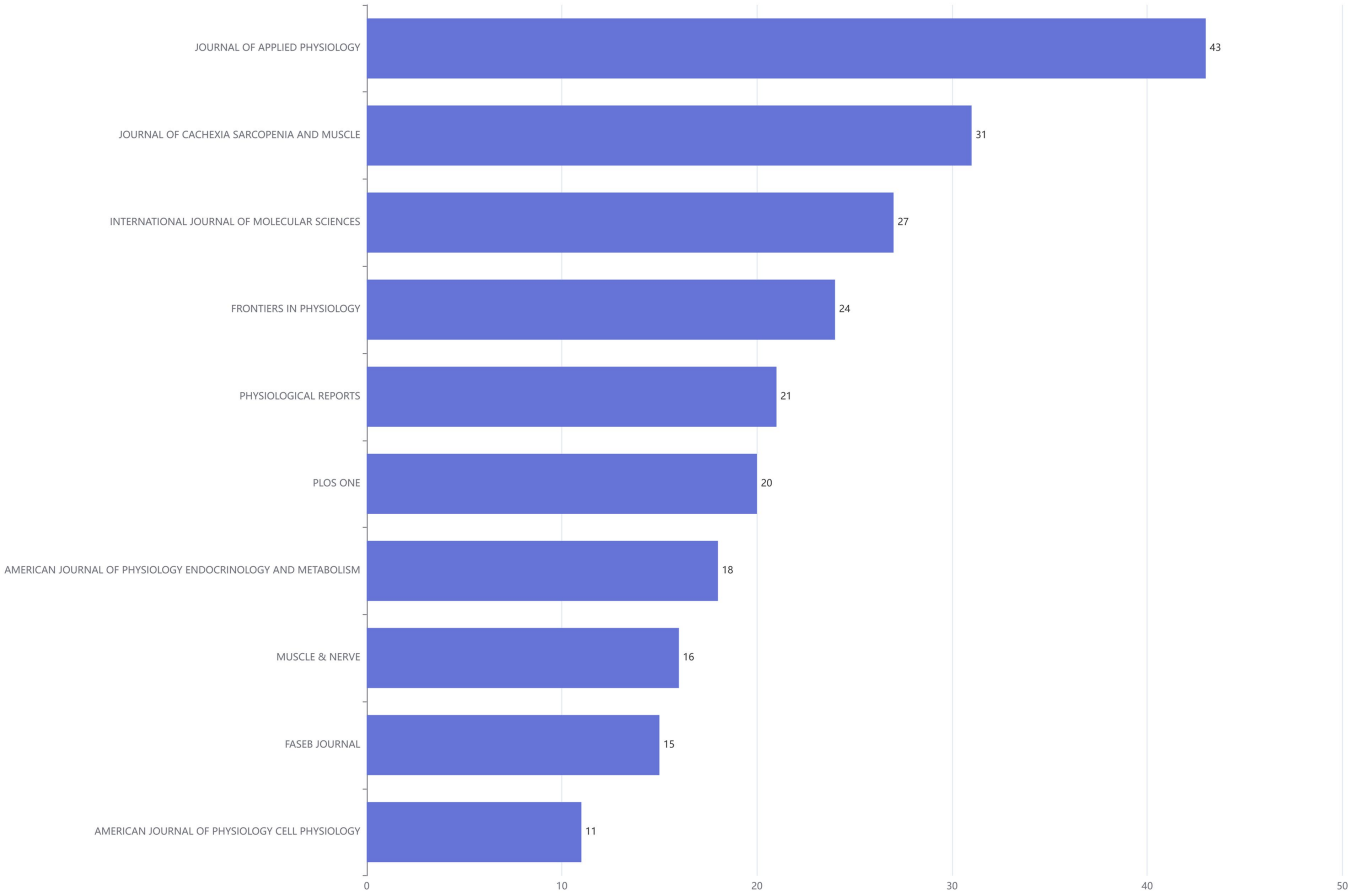
Bar chart of literature sources in the disuse muscle atrophy from January 2010 to December 2024.

**Figure 5:**
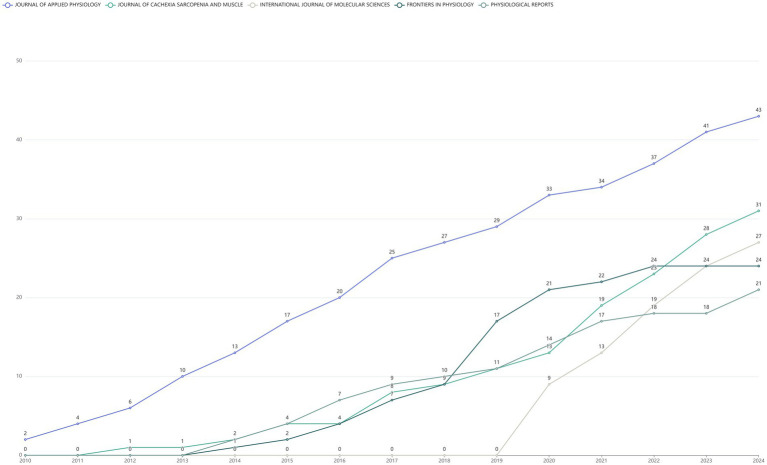
The cumulative publication statistics chart for literature in the disuse muscle atrophy from January 2010 to December 2024, categorized by year of publication.

The top 10 journals ranked by publication volume were shown in the Figure 4. Among them, JOURNAL OF APPLIED PHYSIOLOGY leaded with the highest number of publications, publishing 43 articles. JOURNAL OF CACHEXIA SARCOPENIA AND MUSCLE ranked second with a total of 31 published articles, while INTERNATIONAL JOURNAL OF MOLECULAR SCIENCES took the third spot with 27 articles (Figure 4). In addition, the line graph illustrated the annual publication trends of the top five journals, ranked by cumulative article output, on the DMA. Notably, the JOURNAL OF APPLIED PHYSIOLOGY has consistently published relevant literature each year since 2010 (Figure 5).

### Most relevant authors

**Figure 6:**
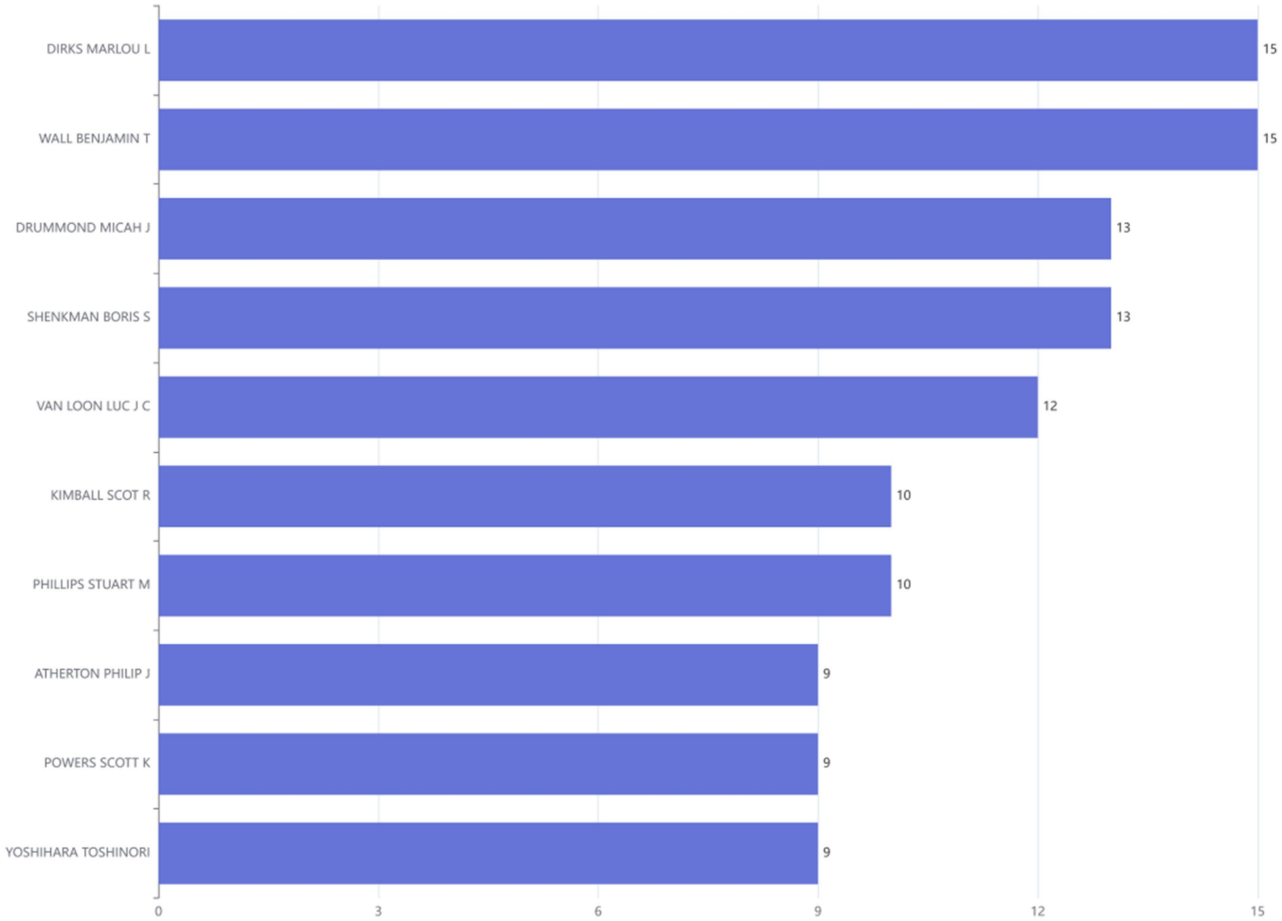
Ranking of authors at the disuse muscle atrophy based on publications from January 2010 to December 2024.

The comprehensive literature review encompassed contributions from a total of 3,401 distinct authors. Through detailed comparative analysis of authors and their affiliated institutions, it was observed that 15.09% of the reviewed publications involved international collaborations. Among the top 10 most prolific authors, DIRKS MARLOU L and WALL BENJAMIN T leaded, each with 15 publications (Figure 6).

### Authors’ production over time

Lotka’s Law illustrated the unequal distribution of academic productivity, where a limited number of prolific authors generated the majority of scholarly output, while most contributors produced only a minimal number of publications. The empirical data demonstrated a notable deviation from the theoretical model: single-author publications (N.Authors = 1) exhibit the highest frequency (Freq = 0.80), significantly exceeding the predicted value of 0.64 (Supplementary Figure S1). This disparity underscored the predominance of authors who published just one article during the study period. Notably, DRUMMOND MICAH J emerged as the most productive contributors in 2023, publishing five articles (Supplementary Figure S2). The substantial output has been instrumental in driving research advancements in the DMA, establishing them as key opinion leaders in this specialized domain.

### Most relevant affiliations

**Figure 7:**
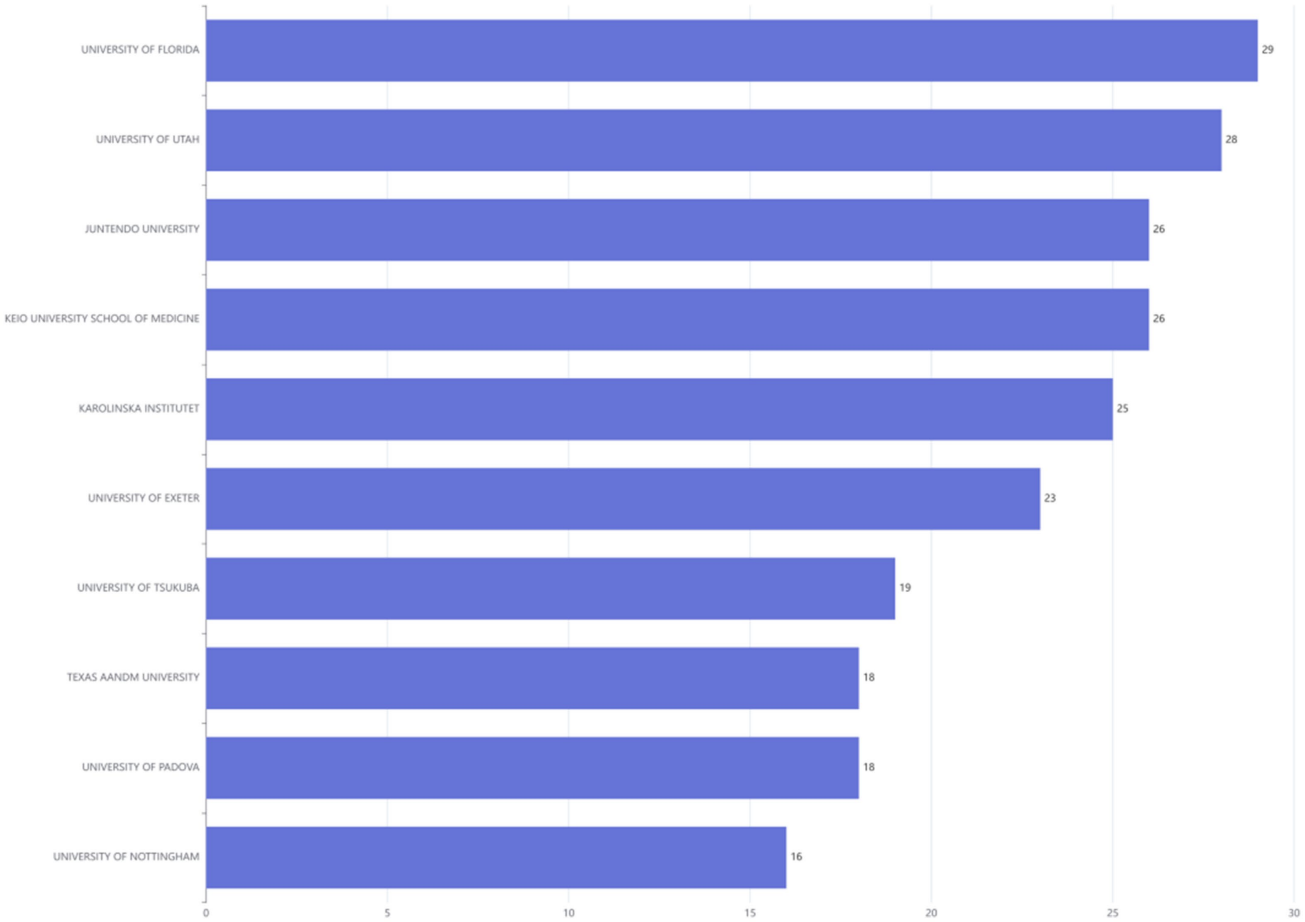
Ranking of institutions publishing in disuse muscle atrophy Research from January 2010 to December 2024.

Figure 7 illustrated the leading research institutions in the DMA, ranked based on their publication output. The UNIVERSITY OF FLORIDA appeared most frequently (29 times each), followed by the UNIVERSITY OF UTAH in second place (28 times).

### Affiliations’ production over time

The line graph illustrated the cumulative publication output of the top five research institutions over different years. Starting from 2019, several institutions have shown a significant increase in their publication numbers (Supplementary Figure S3).

### Corresponding author’s countries

The corresponding authors of the related literature were from 38 countries. Based on the number of publications, the countries of the corresponding authors were ranked. USA leaded with the highest number of articles, followed by the Japan in second place, and China in third place (Supplementary Figure S4).

### Countries’ scientific production

The regional distribution of research output across different countries in the relevant field was illustrated in the figure. The top three countries/regions with the highest number of publications were the USA (347 articles, 22.99%), followed by Japan (301 articles) and China (159 articles) (Supplementary Figure S5).

### Countries’ production over time

The line graph illustrated the cumulative publication output of the top five countries over different years. Starting in 2010, the USA surged to the top in terms of publication volume, reaching a total of 347 articles by December 2024, while Japan ranked second with 301 articles (Supplementary Figure S6). The global citation rankings aligned with the publication rankings, with the USA maintaining its leading position in total citations, accumulating 1892 citations, followed by China with 540 citations (Supplementary Figure S7).

### Most global cited documents

In the relevant literature, the most frequently cited works were as follows: Cohen ShenHav (31) with a total of 489 citations, ranked first; Wang YiChen (32), with 183 citations, took the second position; Narici Marco (33), with 174 citations, was ranked third (Supplementary Figure S8).

### Word cloud

**Figure 8:**
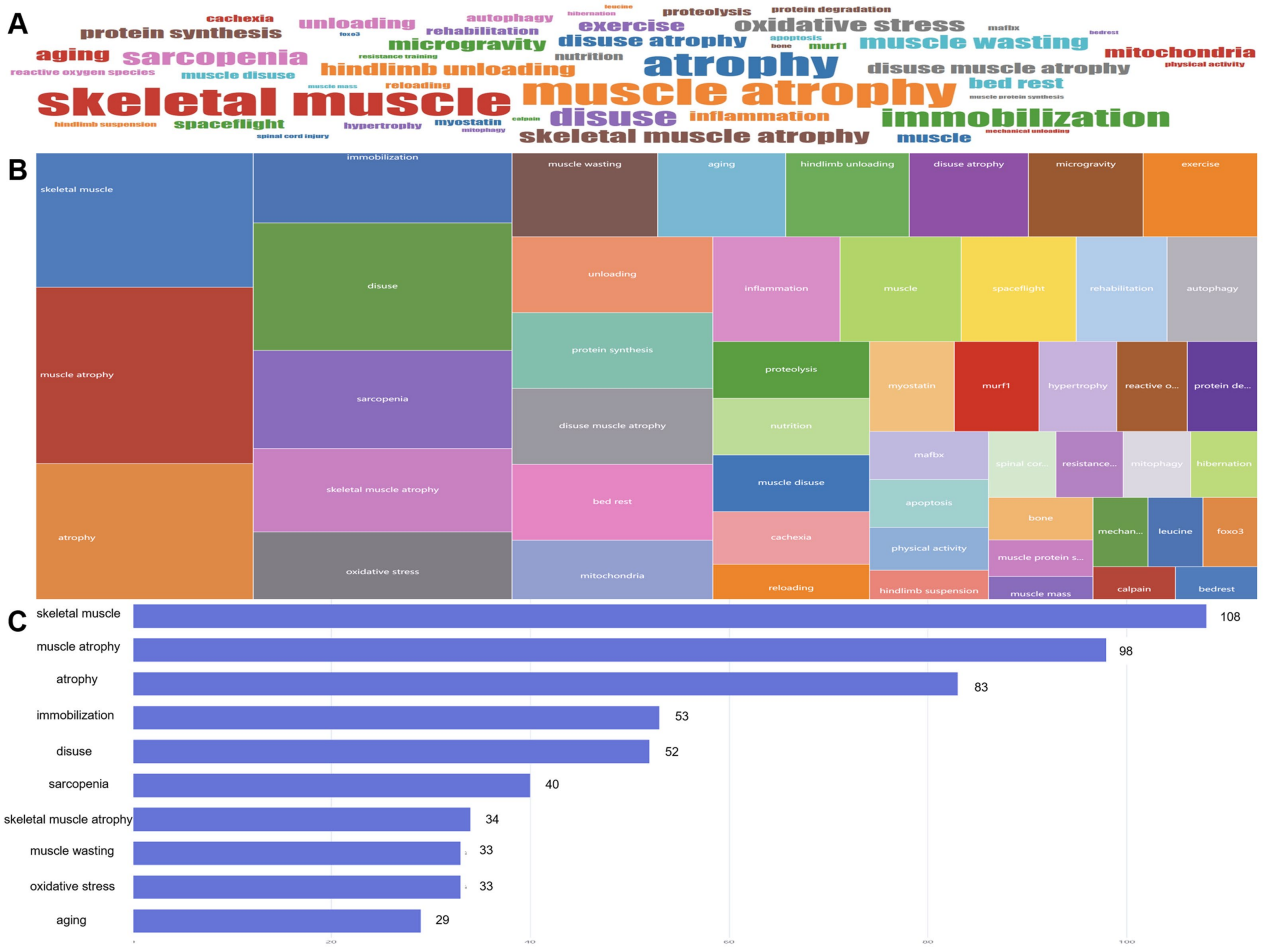
From January 2010 to December 2024, high-frequency keywords in the literature at the disuse muscle atrophy were visualized through a WordCloud **(A)**, TreeMap **(B)**, and bar chart **(C)** illustrating the changes in word frequency.

The analysis of author keywords in the article was conducted to evaluate various themes in the DMA. A word cloud and tree map were used to visualize the 50 most frequently occurring author keywords (Figures 8A,B). The most prominent keywords included “skeletal muscle” (108 times), “muscle atrophy” (98 times), “atrophy” (23 times), “immobilization” (53 times), and “disuse” (52 times) (Figure 8C).

### Words’ frequency over time

When analyzing the frequency of keywords in the DMA literature over the years, it was evident that key terms related to skeletal muscle, muscle atrophy, immobilization, and sarcopenia were prominently featured. In addition, terms such as OXIDATIVE STRESS and AGING have consistently been the focus of research since 2012 (Supplementary Figure S9).

### Trend topics

**Figure 9:**
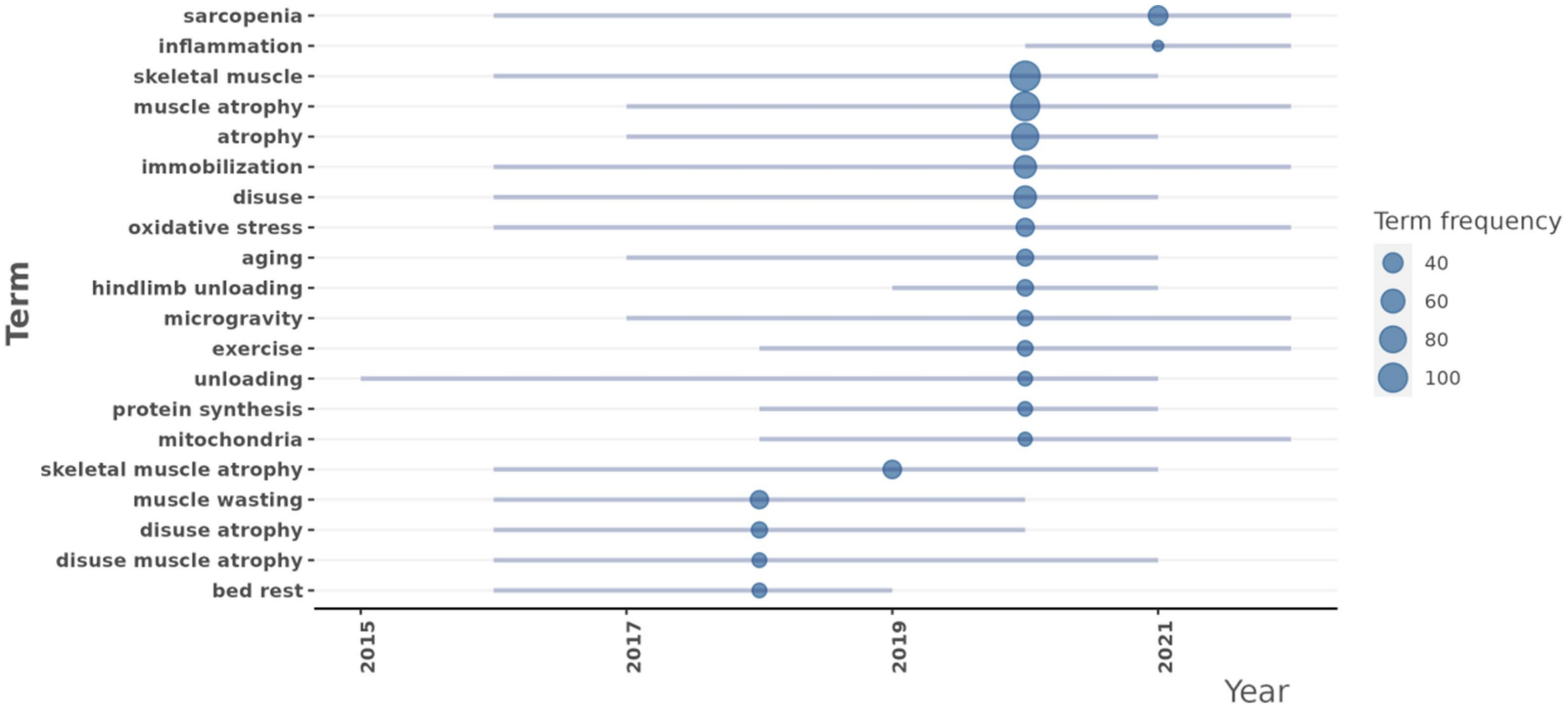
Research topic trends at the disuse muscle atrophy from January 2010 to December 2024.

A trend analysis was conducted on scientific articles addressing the DMA. By examining the literature data from this period, the primary research themes and their evolutionary trends were identified (Figure 9). In this study, we focused on highlighting relatively specific or distinctive keywords to avoid redundancy, as many commonly occurring terms were less informative or too general. Therefore, only the more representative or unique keywords were listed and discussed in the text. The analysis revealed several specific key research topics that have shown significant development trends in recent years:

Oxidative stress (frequency: 33): Research in this area began to increase in 2016, with a median year of 2020, and maintained a high research frequency through 2022. This indicated that oxidative stress has remained a consistently focused area in the DMA research.

Aging (frequency: 29): Research in aging saw a rapid rise starting in 2017, with a median year of 2021, and remained active through 2021.

Exercise (frequency: 26): Research in exercise significantly increased from 2018, with a median year of 2020, and continued actively through 2022.

Protein synthesis (frequency: 24): Research in protein synthesis began to grow in 2018, with a median year of 2020, and was still ongoing in 2021.

Mitochondria (frequency: 23): Research related to mitochondria started to increase in 2018, with a median year of 2020, and remained active through 2022.

Inflammation (frequency: 21): Research related to inflammation started to increase in 2020, with a median year of 2021, and remained active through 2022.

### Clustering by coupling

After conducting a coupling cluster analysis on scientific articles related to the DMA, 5 major clusters were identified, each with an impact score of 1.000. These clusters exhibited distinct research focuses and characteristics (Supplementary Figure S10). The largest cluster, comprising 74 articles, was primarily labeled with “oxidative stress - conf 76.5%,” highlighting its dominant position. The centrality score of 0.4673 underscored its importance within the overall network.

### Co-occurence network

The keyword contribution network analysis revealed that nodes represented keywords, and the connections between nodes indicated co-occurrence or citation relationships. Key terms related to skeletal muscle, muscle atrophy, immobilization, and sarcopenia were prominently highlighted (Supplementary Table S1; Supplementary Figures S11A–C). In the co-occurrence network, “oxidative stress” emerged as one of the most significant nodes, with a betweenness centrality (21.4904), a relatively high closeness centrality (0.0125), and a notable PageRank value (0.0303). This highlighted “oxidative stress” as a pivotal bridge connecting diverse research themes and solidified its central role in the network. Additionally, “exercise” and “aging” were also key nodes, with betweenness centrality values of 16.8278 and 13.5887, respectively, underscoring their critical mediating roles (Supplementary Table S1). The density map visually represented the interactions between keywords, with darker areas indicating higher co-occurrence frequencies or stronger relational densities. In this map, skeletal muscle atrophy appeared in darker regions, signifying frequent interactions and discussions between these topics and reflecting current research hotspots (Supplementary Figure S14B). The degree distribution plot illustrated the number of connections each node has, with skeletal muscle atrophy prominently featured (Supplementary Figure S11C). This aligned with their central positions in the network and indicated their extensive connections with other nodes, marking them as focal keywords in the research.

### Thematic map

By integrating Callon’s centrality and density metrics, the research topics related to the DMA were primarily divided into 12 clusters (Supplementary Figure S12A,B). In addition to commonly keywords, main specific or distinctive clusters were as followings:

Murf1 (Centrality: 1.9492, Density: 44.6763, Centrality Rank: 10): This cluster hold a significant central position in the network, indicating strong connections to other topics and a high level of internal coherence. Its high centrality rank confirmed that Murf1 remained a core theme in the field.
Soleus muscle (Centrality: 1.1231, Callon Density: 39.6241, Centrality Rank: 9): This cluster shared a similar centrality to murf1, suggesting that soleus muscle also played a central role in the research network. Its higher Callon density reflected even greater internal consistency.
Foxo3 (Centrality: 0.55, Density: 28.611, Centrality Rank: 8): This cluster demonstrated a notable presence in the network, with a moderate level of centrality and a relatively high density, indicating its importance in the field.
Ros (Centrality: 0.3333, Density: 33.3333, Centrality Rank: 7): This cluster also hold a significant position, with a slightly lower centrality.
Glucocorticoids (Centrality: 0.2222, Density: 33.3333, Centrality Rank: 6): This cluster, while having a lower centrality, still showed a significant level of internal coherence, indicating its growing importance in the domain.

### Co-citation network

A co-citation network analysis was performed to map the core literature on the DMA in 2008 & 2013 and their positions within the academic network (Supplementary Figure S13). The study by Bodine SC (34) and Glover EI (35) stood out as the most influential paper, with its high betweenness centrality and PageRank score indicating its pivotal role in the research community. These findings not only highlight key contributors at the intersection of AI and orthopedics but also shed light on the interconnections and distribution of influence across research themes.

### Collaboration network

A thorough examination of global literature indicated that 3,401 authors have contributed to this field of research. A collaborative network was formed, consisting of authors who have published a minimum of two papers. As illustrated in the co-authorship network diagram, Atherton Philip J stood out as the most influential researcher, with his high betweenness centrality and PageRank scores highlighting his pivotal role within the research community. Furthermore, other researchers such as Shenkman Boris S, Dirks Marlou L, Wall Benjamin T, and Drummond Micah J, though less prominent, still occupied significant positions in the network, demonstrating their active involvement and impact in the co-authorship network of the literature (Supplementary Figure S14).

### Countries’ collaboration world map

When analyzing the patterns and trends in global scientific research collaboration, it became evident that the most frequent occurrence of cooperation was nine. We observed that the United States maintained extensive collaborative relationships with multiple countries, including China, Japan, Sweden, and Brazil. Notably, the frequency of cooperation with China and Japan stood at seven instances each. Additionally, European countries such as Italy, Germany, and Denmark exhibited a strong tendency to collaborate with one another (Supplementary Table S2; Supplementary Figure S15).

### Literature recommendation

By evaluating global academic literature, 10 influential papers were identified based on their impact factor (Table 1). Furthermore, 10 frequently cited papers were selected according to their citation metrics (Table 2).

**Table 1:** High-scoring recommended literature based on the impact factor criteria.

Rank	Article title	Release time	Journal	Impact factor
1	Muscle wasting in disease: molecular mechanisms and promising therapies.	2015/1/1	NATURE REVIEWS DRUG DISCOVERY	122.7
2	Activation of calcium signaling through Trpv1 by nNOS and peroxynitrite as a key trigger of skeletal muscle hypertrophy.	2012/12/4	NATURE MEDICINE	58.7
3	Novel treatment strategies for chronic kidney disease: insights from the animal kingdom.	2018/1/16	NATURE REVIEWS NEPHROLOGY	28.6
4	Regulation of muscle atrophy-related genes by the opposing transcriptional activities of ZEB1/CtBP and FOXO3.	2018/10/11	NUCLEIC ACIDS RESEARCH	16.6
5	Implantable Epigallocatechin Gallate Sustained-Release Nanofibers for the Prevention of Immobilization-Induced Muscle Atrophy.	2023/12/24	ACS NANO	15.8
6	Losartan restores skeletal muscle remodeling and protects against disuse atrophy in sarcopenia.	2011/5/13	SCIENCE TRANSLATIONAL MEDICINE	15.8
7	miR-29b contributes to multiple types of muscle atrophy.	2017/5/26	NATURE COMMUNICATIONS	14.7
8	Disruption of bone and skeletal muscle in severe burns.	2015/8/15	BONE RESEARCH	14.3
9	Piezo1: opening the way to preventing muscle atrophy.	2022/5/16	JOURNAL OF CLINICAL INVESTIGATION	13.3
10	Models of accelerated sarcopenia: critical pieces for solving the puzzle of age-related muscle atrophy.	2010/5/5	AGEING RESEARCH REVIEWS	12.5

**Table 2:** Identify highly cited literature based on citation count.

Rank	Article title	Release time	Journal	Impact factor	Citation count
1	Muscle wasting in disease: molecular mechanisms and promising therapies.	2015/1/1	NATURE REVIEWS DRUG DISCOVERY	122.7	489
2	Mechanisms for fiber-type specificity of skeletal muscle atrophy.	2013/3/16	CURRENT OPINION IN CLINICAL NUTRITION AND METABOLIC CARE	3	183
3	Impact of sedentarism due to the COVID-19 home confinement on neuromuscular, cardiovascular and metabolic health: Physiological and pathophysiological implications and recommendations for physical and nutritional countermeasures.	2020/5/13	EUROPEAN JOURNAL OF SPORT SCIENCE	2.4	174
4	Skeletal muscle atrophy during short-term disuse: implications for age-related sarcopenia.	2013/8/17	AGEING RESEARCH REVIEWS	12.5	157
5	Disuse-induced muscle wasting.	2013/6/27	INTERNATIONAL JOURNAL OF BIOCHEMISTRY & CELL BIOLOGY	3.4	150
6	FoxO transcription factors: their roles in the maintenance of skeletal muscle homeostasis.	2013/11/16	CELLULAR AND MOLECULAR LIFE SCIENCES	6.2	131
7	Activation of calcium signaling through Trpv1 by nNOS and peroxynitrite as a key trigger of skeletal muscle hypertrophy.	2012/12/4	NATURE MEDICINE	58.7	122
8	Stress-induced skeletal muscle Gadd45a expression reprograms myonuclei and causes muscle atrophy.	2012/6/14	JOURNAL OF BIOLOGICAL CHEMISTRY	4	121
9	Losartan restores skeletal muscle remodeling and protects against disuse atrophy in sarcopenia.	2011/5/13	SCIENCE TRANSLATIONAL MEDICINE	15.8	118
10	Uraemic sarcopenia: aetiology and implications.	2013/4/30	NEPHROLOGY, DIALYSIS, TRANSPLANTATION: OFFICIAL PUBLICATION OF THE EUROPEAN DIALYSIS AND TRANSPLANT ASSOCIATION - EUROPEAN RENAL ASSOCIATION		116

## Discussion

Our findings indicated that DMA has gradually become a research hotspot in skeletal muscle science, with publication numbers peaking in 2020. The University of Utah played a significant role in this field. Journals such as the Journal of Applied Physiology, Journal of Cachexia, Sarcopenia and Muscle, and International Journal of Molecular Sciences led with the highest number of publications. Researchers like Dirks MarLou L, Wall Benjamin T, and Drummond Micah J made substantial contributions to this field. The United States, China, and Japan were major contributors. Works by Cohen ShenHav (31), Wang YiChen (32), and Narici Marco (33) were frequently cited. Key terms such as “oxidative stress,” “aging,” “exercise,” “protein synthesis,” “mitochondria,” “inflammation,” “Murf1,” “soleus muscle,” “Foxo3,” “ROS,” and “glucocorticoids” emerged as popular topics. These research hotspots may play a crucial role in the molecular mechanisms underlying disuse-induced muscle atrophy.

Our results showed that although the volume of publications has shown a upward trend, citation counts have been gradually decreasing. The apparent drop in average citation counts for recent years was likely due to the citation time lag—newly published articles have had less time to accumulate citations compared to older publications. This was a well-known limitation in bibliometric studies and did not necessarily indicate reduced impact or interest.

Our analysis indicated a noticeable increase in publications on DMA starting around 2020. This may be partly attributed to the heightened clinical and research interest in muscle loss and physical deconditioning associated with prolonged immobilization during the COVID-19 pandemic. We re-examined the keyword co-occurrence data and identified the term of COVID-19, with four times. These findings suggest that COVID-19-related research may have contributed to the increased focus on DMA. However, there was no direct relationship between keywords and COVID-19.

## Future directions

Among the topics identified in this study, certain frequently occurring keywords, such as “muscle atrophy,” were highly general and broadly represented across the literature. Although these terms reflected overall research interest, they tended to lack specificity and were less useful for guiding future, targeted investigations. Consequently, a subset of topics, despite being less frequent, provided more distinctive or emerging perspectives with greater potential to shape novel research directions.

Oxidative stress played a pivotal role in the onset and progression of disuse-induced muscle atrophy. It occured when the levels of reactive oxygen species (ROS) exceeded the capacity of the antioxidant defense system, leading to cellular damage. Research indicates that in disuse muscle atrophy, alterations in mitochondrial phospholipids were considered a potential source of ROS, contributing to muscle dysfunction (8). Furthermore, oxidative stress exacerbated muscle atrophy by disrupting mitochondrial dynamics, including fusion and fission processes (9). Additionally, oxidative stress promoted muscle protein degradation by activating the ubiquitin-proteasome pathway and autophagy. Studies have shown that oxidative stress upregulated the expression of muscle-specific E3 ubiquitin ligases such as MAFbx and MuRF1, accelerating protein breakdown (10). Lastly, oxidative stress can enhance the production of inflammatory factors like TNF-*α* and IL-6, which not only directly damaged muscle cells but also indirectly accelerated atrophy by activating protein degradation pathways (11). In summary, oxidative stress contributed to disuse muscle atrophy through multiple mechanisms, and understanding these pathways was crucial for developing effective therapeutic strategies.

Aging significantly influenced the onset and progression of disuse-induced muscle atrophy. As individuals age, the gradual decline of physiological systems made maintaining muscle mass increasingly difficult, partly due to anabolic resistance, which exacerbates muscle loss (12). Disuse atrophy, often resulting from reduced physical activity, illness, immobilization, or hospitalization, accelerated this process (12). The underlying mechanisms of disuse atrophy included anabolic resistance, inflammation, protein homeostasis imbalance, and mitochondrial dysfunction, all contributing to a negative net protein balance and subsequent muscle loss (13). Research indicated that mitochondrial dysfunction was a common factor in skeletal muscle atrophy associated with disease, aging, and prolonged inactivity (14). Impaired mitochondrial function was a primary cause of muscle atrophy, as it can lead to oxidative stress and activate protein degradation systems, both of which were critical in disuse atrophy (15). Furthermore, studies have shown that the mTORC1 signaling pathway was activated during aging and co-localizes with fiber damage. This activation increased mitochondrial oxidative stress and the expression of catabolic genes, leading to muscle fiber damage and loss (16). Therefore, aging can affect the development of disuse-induced muscle atrophy through multiple mechanisms, including anabolic resistance, mitochondrial dysfunction, and aberrant activation of the mTORC1 signaling pathway.

Exercise played a pivotal role in the onset and progression of disuse-induced muscle atrophy. Research indicated that resistance training is an effective strategy to counteract this condition. Resistance training significantly stimulated muscle protein synthesis, thereby mitigating muscle loss. This form of exercise included traditional weightlifting, bodyweight exercises, and both low- and high-intensity resistance training. When combined with adequate dietary protein, resistance training can accelerate recovery from disuse events in older adults, reduce frailty, and enhance mobility (12). Moreover, exercise preconditioning has been shown to mitigate the effects of disuse-induced muscle atrophy. Studies suggested that exercise preconditioning can alleviate muscle wasting caused by disuse and provide potential mechanisms to prevent muscle depletion. This protective effect was particularly crucial in aging muscles, as older individuals were more susceptible to disuse atrophy (17). Furthermore, exercise can delay muscle atrophy by modulating microRNAs (miRNAs) within the muscle. miRNAs played a significant role in exercise-induced changes in muscle mass, composition, and function. By regulating miRNAs, exercise may help slow the progression of muscle atrophy in older adults (18). In summary, physical activity was essential for both preventing and treating disuse-induced muscle atrophy, supporting muscle health and function through multiple mechanisms.

Protein synthesis was essential in disuse-induced muscle atrophy, and a deeper understanding of its mechanisms can contribute to the development of more effective therapeutic strategies. Research showed that muscle disuse led to a rapid decline in muscle mass, primarily due to a reduction in muscle protein synthesis (MPS) (19). During disuse-induced muscle atrophy, dysregulation of the eEF2k/eEF2 pathway was considered a primary mechanism responsible for the decreased rate of protein synthesis (20). Additionally, the mTORC1 signaling pathway played a significant role in regulating muscle protein synthesis and muscle size, especially in atrophy caused by denervation (21). In the early stages of disuse-induced muscle atrophy, the reduction in protein synthesis was closely associated with muscle mass loss. Using systems biology approaches, researchers have identified molecular networks and key candidate molecules quantitatively linked to disuse-induced muscle atrophy and the extent of MPS decline (19). These findings provided a strong foundation for advancing our understanding of the mechanisms underlying short-term disuse-induced muscle atrophy and for developing therapeutic interventions.

The role of mitochondria in disuse-induced muscle atrophy was a complex and significant area of research. Disuse-induced muscle atrophy, characterized by a decline in muscle mass and function due to inactivity, was closely associated with alterations in mitochondrial function (13). Studies suggested that changes in mitochondrial phospholipids may be a key source of oxidative stress in disuse-induced muscle atrophy, which in turn led to muscle dysfunction (8). Moreover, mitochondrial dysfunction was recognized as a major contributor to muscle atrophy under various conditions, including disease, aging, and prolonged inactivity (14). In the context of disuse-induced muscle atrophy, increased electron leakage and oxidative stress in mitochondria may result from changes in mitochondrial membrane lipids. This oxidative stress not only caused cellular damage but also activated signaling pathways that further exacerbated muscle atrophy (8). Additionally, mitochondrial dynamics and mitophagy played crucial roles in maintaining muscle health, particularly during aging, where the decline in mitochondrial function was closely linked to the loss of muscle mass (22). In summary, the involvement of mitochondria in disuse-induced muscle atrophy extended beyond reduced energy supply and includes oxidative stress, activation of signaling pathways, and organelle quality control. Understanding these mechanisms was essential for developing effective prevention and treatment strategies.

The role of inflammation in disuse muscle atrophy was significant, and modulating inflammatory responses may provide new strategies for prevention and treatment. Research suggested that inflammatory reactions were among the older adults triggers of muscle atrophy. In models of disuse muscle atrophy, levels of inflammatory factors were markedly elevated, potentially leading to increased muscle protein breakdown and accelerating muscle wasting (23). Additionally, inflammatory signaling pathways were intricately linked to muscle metabolism and function. In the older adults, altered inflammatory responses can impair muscle regeneration and recovery, exacerbating the severity of muscle atrophy (24). Studies have also demonstrated that suppressing inflammation can effectively mitigate disuse muscle atrophy. For example, anti-inflammatory drugs such as aspirin can reduce denervation-induced muscle atrophy by modulating relevant signaling pathways (25). Future research can delve deeper into the specific mechanisms connecting inflammation and muscle atrophy to develop more effective therapeutic approaches.

Glucocorticoids played a significant role in the development and progression of disuse muscle atrophy. Elevated levels of glucocorticoids can impair muscle mass and function, leading to muscle atrophy (26). Research indicated that glucocorticoid-induced muscle atrophy contributed to fatigue during daily activities, such as climbing stairs or brisk walking, thereby reducing physical activity capacity 26]. Moreover, glucocorticoids regulated muscle atrophy by activating autophagy pathways. Sirtuin 2 (SIRT2) was believed to influence autophagy signaling and affect glucocorticoid-induced muscle atrophy. Studies have shown that activating SIRT2 can protect muscle cells from glucocorticoid-induced atrophy by suppressing the autophagy system (27). In terms of treatment, adequate nutrition was crucial for limiting muscle wasting and enhancing muscle mass recovery. Nutrients such as branched-chain amino acids, *β*-hydroxy β-methylbutyrate, omega-3 fatty acids, and various vitamins and their derivatives have been reviewed for their potential roles in preventing and mitigating glucocorticoid-induced muscle atrophy (26). These findings provided valuable insights into the mechanisms by which glucocorticoids contributed to disuse muscle atrophy and offered direction for future therapeutic strategies.

The interaction between MURF1 and FOXO3 in disuse atrophy and their specific manifestations in the soleus muscle provided important insights into the molecular mechanisms of muscle atrophy. FOXO3, a transcription factor, has been shown to play a crucial role in the process of muscle atrophy. Previous studies indicated that FOXO3 promoted muscle atrophy by inducing a group of proteins known as atrogenes, including MURF1 and Atrogin-1 (28). These proteins were part of the ubiquitin-proteasome and autophagy-lysosomal systems, which were involved in protein degradation and organelle clearance, leading to a reduction in muscle proteins and muscle fiber atrophy. Furthermore, MURF1, acting as an E3 ubiquitin ligase, directly participated in the degradation of muscle proteins. The expression of MURF1 significantly increased under conditions of muscle atrophy, and this increase was closely linked to the activity of FOXO3 (29). In models of disuse-induced muscle atrophy, FOXO3 regulated MURF1 expression, promoting the degradation of muscle proteins and exacerbating muscle atrophy. In the Soleus muscle, the interaction between MURF1 and FOXO3 was equally important. The Soleus muscle, which was predominantly composed of slow-twitch fibers, was particularly susceptible to disuse atrophy. During the disuse atrophy of the Soleus muscle, the activity of FOXO3 increased, leading to an upregulation of MURF1 expression, which in turn accelerated muscle atrophy (30). These studies not only helped to elucidate the pathophysiological mechanisms of muscle atrophy but also offered potential targets for developing therapeutic strategies against it.

## Limitations

This study has several limitations. Firstly, the data were sourced exclusively from the PubMed database, which may have led to the omission of relevant articles published in other databases. Secondly, although the research strategy was designed to gather data as comprehensively as possible, it could not guarantee that all included articles were entirely relevant. Nevertheless, a few irrelevant or missed articles are unlikely to significantly affect the overall estimation of research trends.

## Conclusion

This study highlighted an increasing emphasis on molecular mechanisms, such as oxidative stress, aging, protein synthesis, mitochondrial function, and inflammation. Murf1 and Foxo3 played crucial roles in the process of DMA. Both exercise and glucocorticoids were primary methods for enhancing muscle recovery. The soleus muscle was the primary focus in DMA studies. This study provided a valuable reference for guiding future research and optimizing potential intervention strategies for DMA.

## Data Availability

The raw data supporting the conclusions of this article will be made available by the authors, without undue reservation.

## Footnotes

^1^ https://www.bibliometrix.org
