# Supplementary material for: Trends and frontiers in disuse muscle atrophy research
Source: Front Public Health. 2025 Nov 17;13:1611571. doi: 10.3389/fpubh.2025.1611571 (PMC12667248; doi:10.3389/fpubh.2025.1611571)
Supplement: Supplementary file 1 [file Table_1.docx]

**Supplementary Materials**


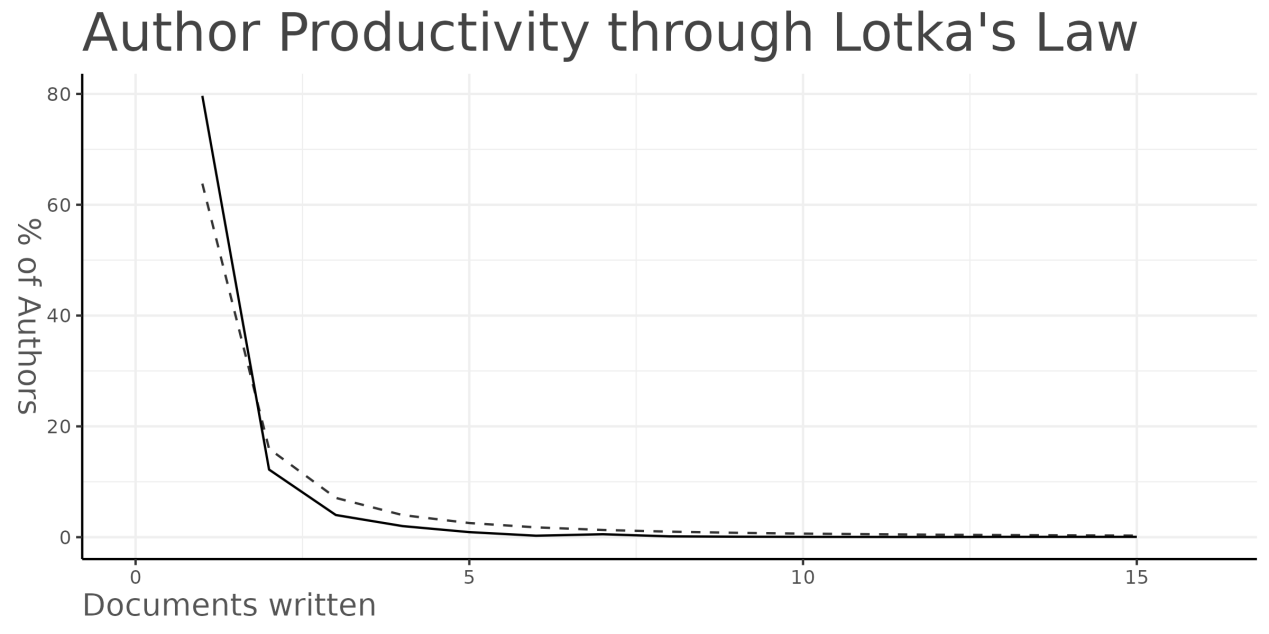


**Fig. S1** Evaluation of Author Productivity in the Disuse Muscle Atrophy from January 2010 to December 2024 Based on Lotka's Law.

**
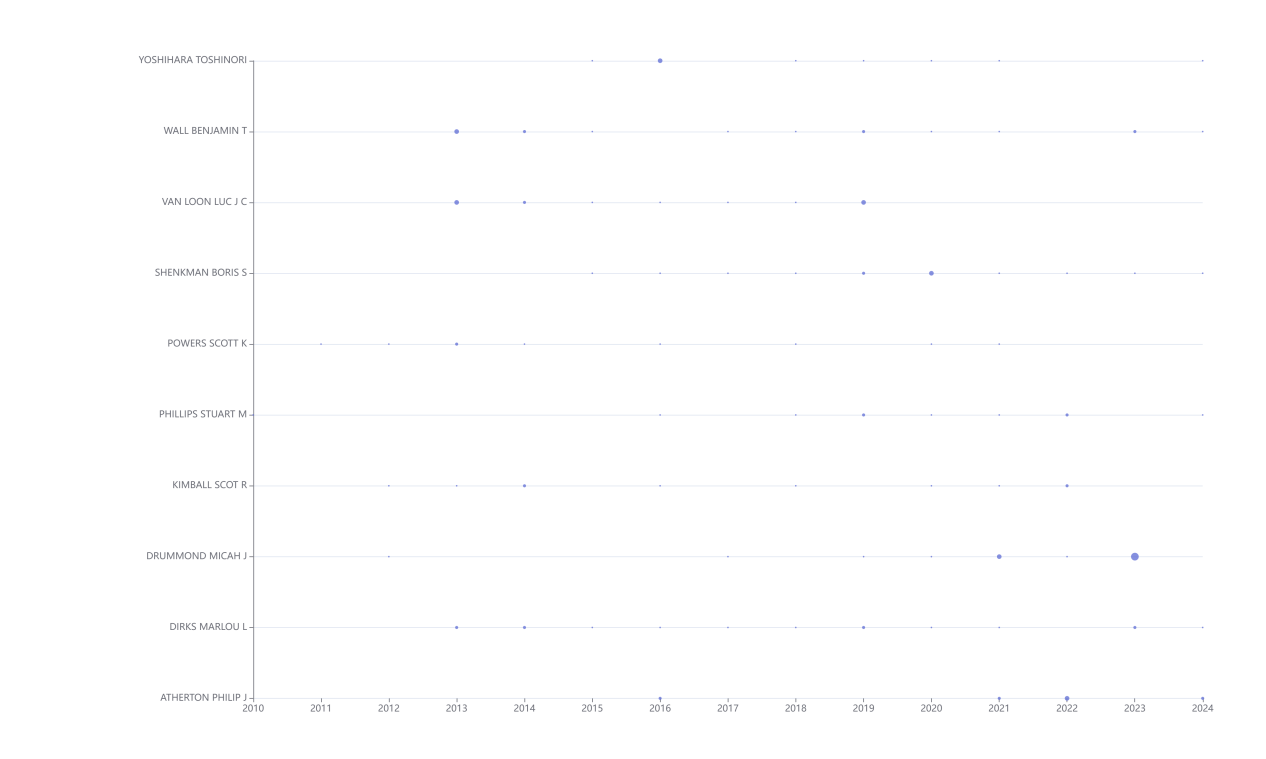
Fig. S2** Publications by Authors in the the Disuse Muscle Atrophy from January 2010 to December 2024, Categorized by Year.

**
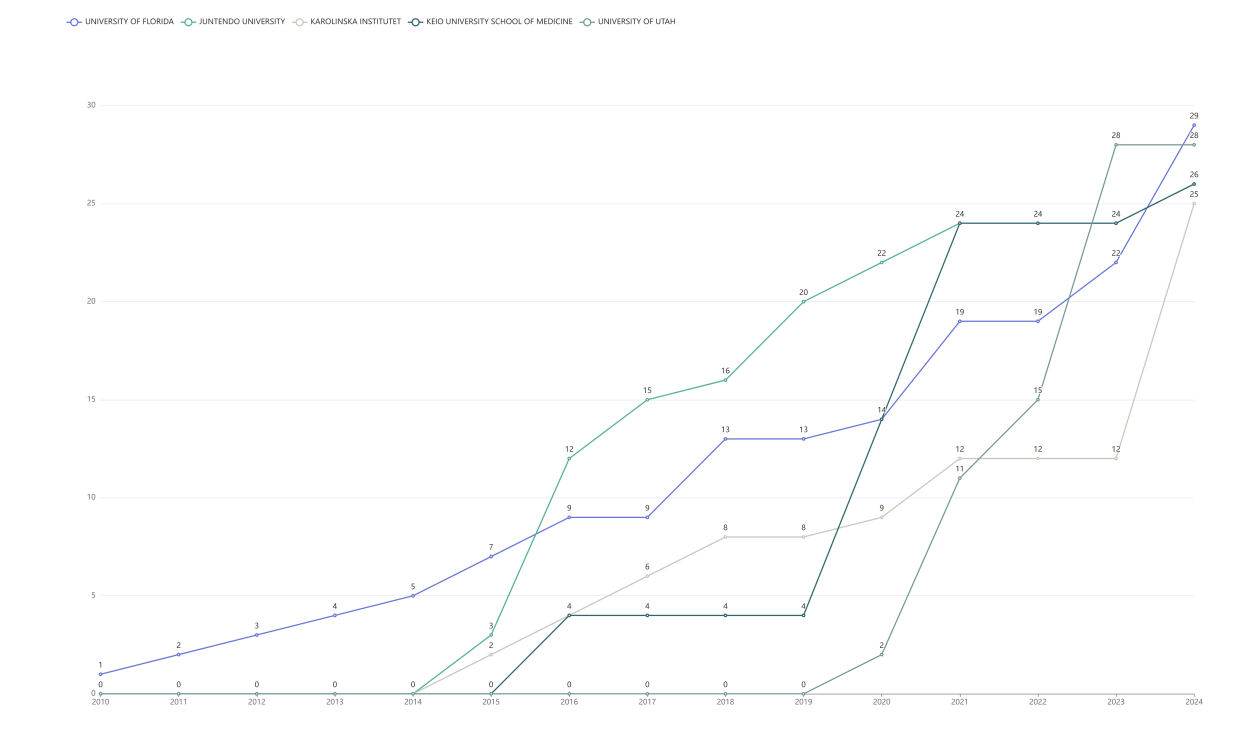
**

**Fig. S3** Timeline of Research Output from Institutions in the Disuse Muscle Atrophy, January 2010 to December 2024.

**
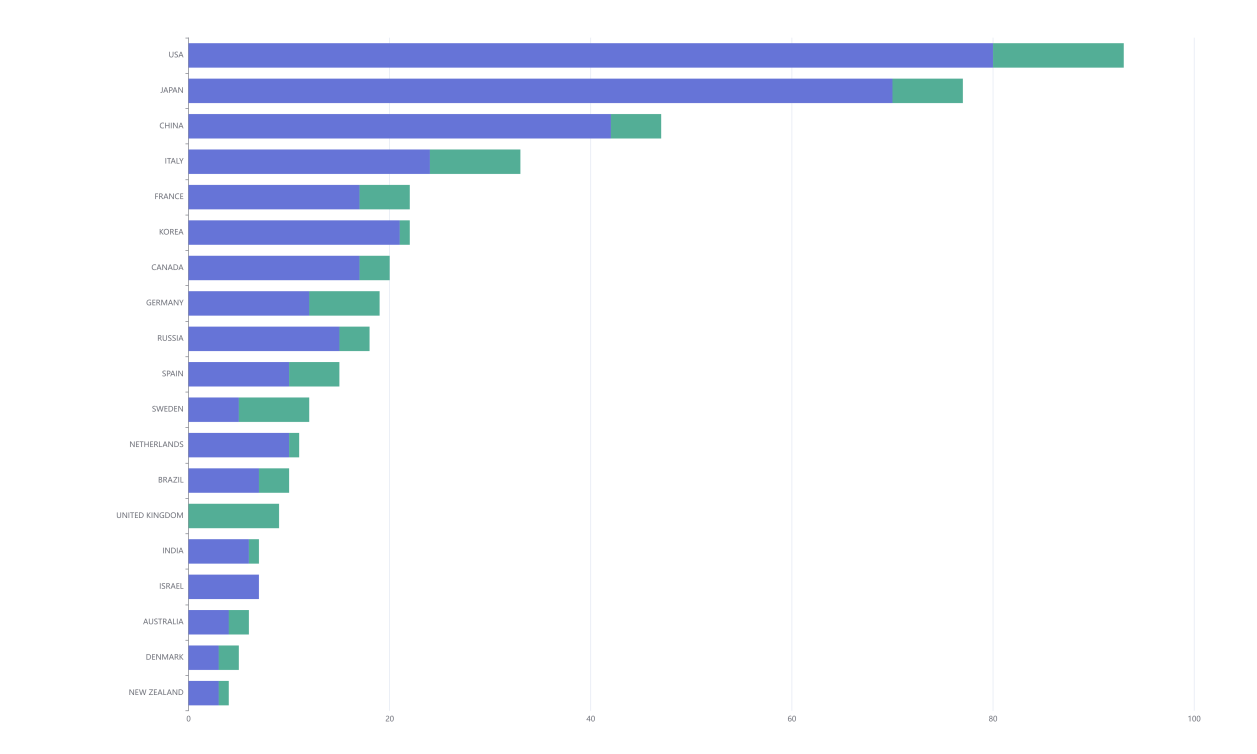
**

**Fig. S4** Ranking of Corresponding Authors' Countries and International Collaboration Ratios in the Field of the Disuse Muscle Atrophy Research from January 2010 to December 2024.

**
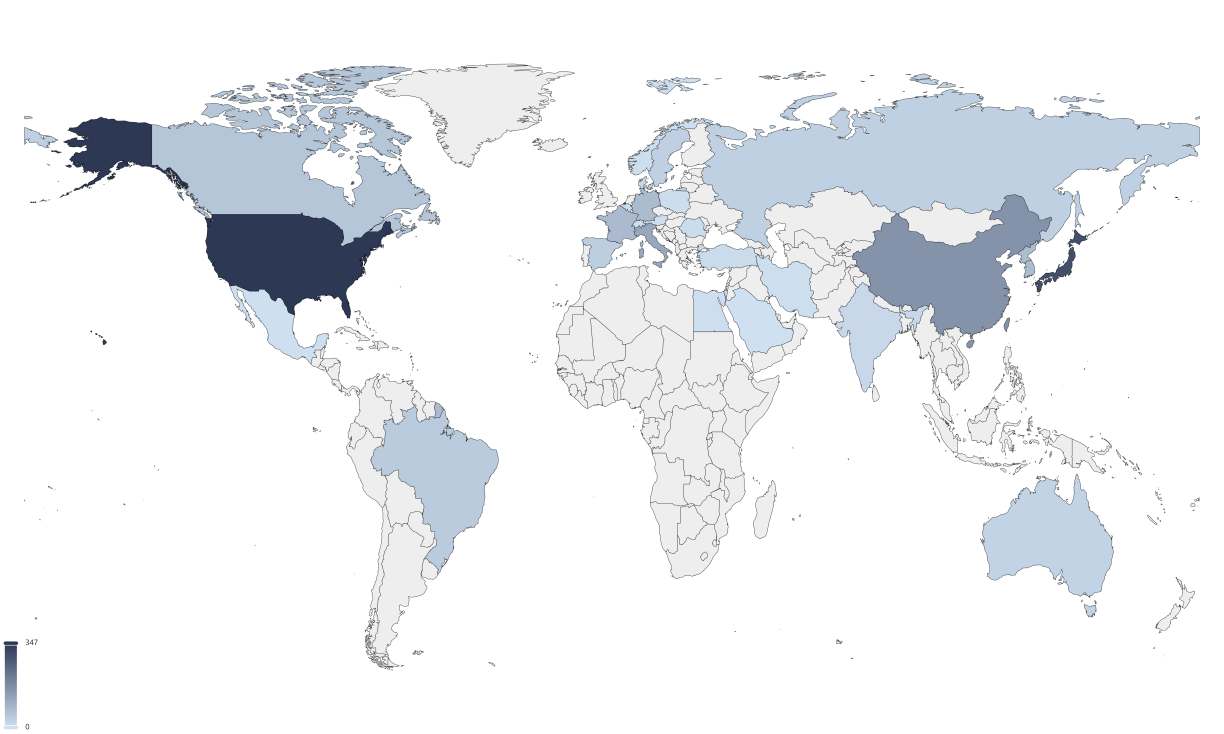
**

**Fig. S5** The regional distribution map of scientific output in the Disuse Muscle Atrophy from January 2010 to December 2024.

**
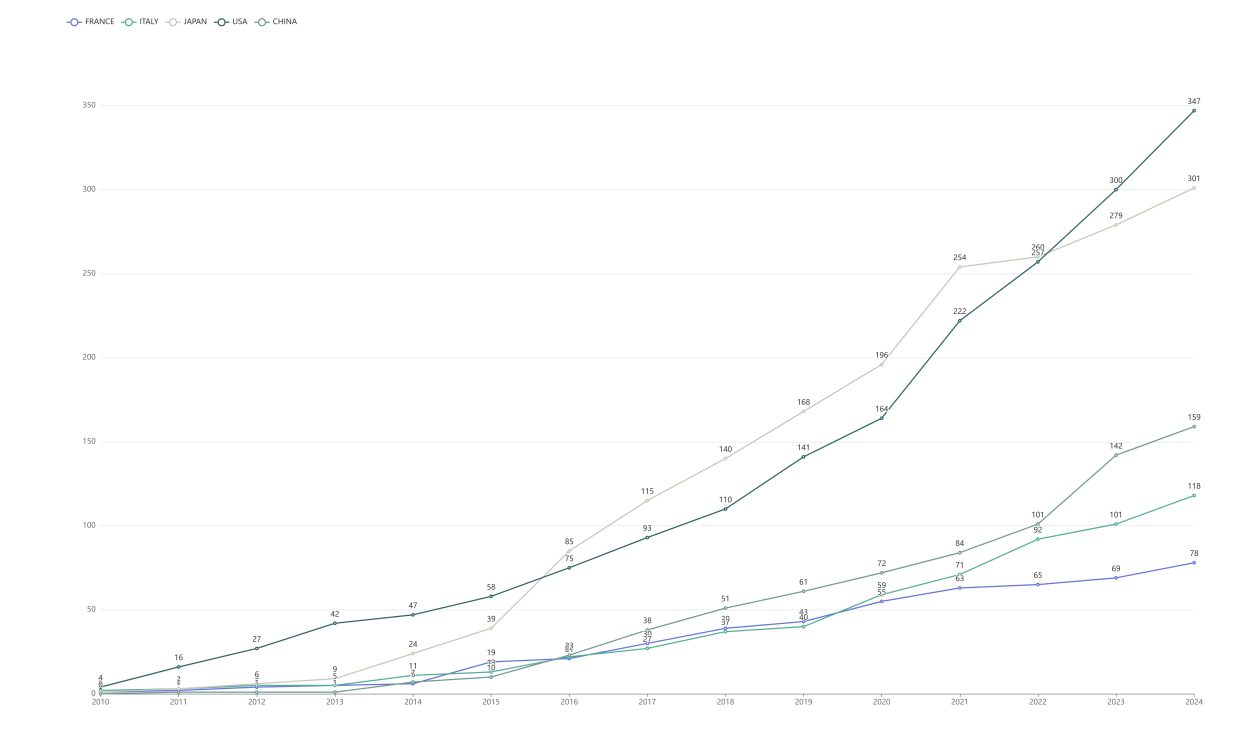
Fig. S6** Chart of Output Over Time by Country in the Disuse Muscle Atrophy from January 2010 to December 2024.

**
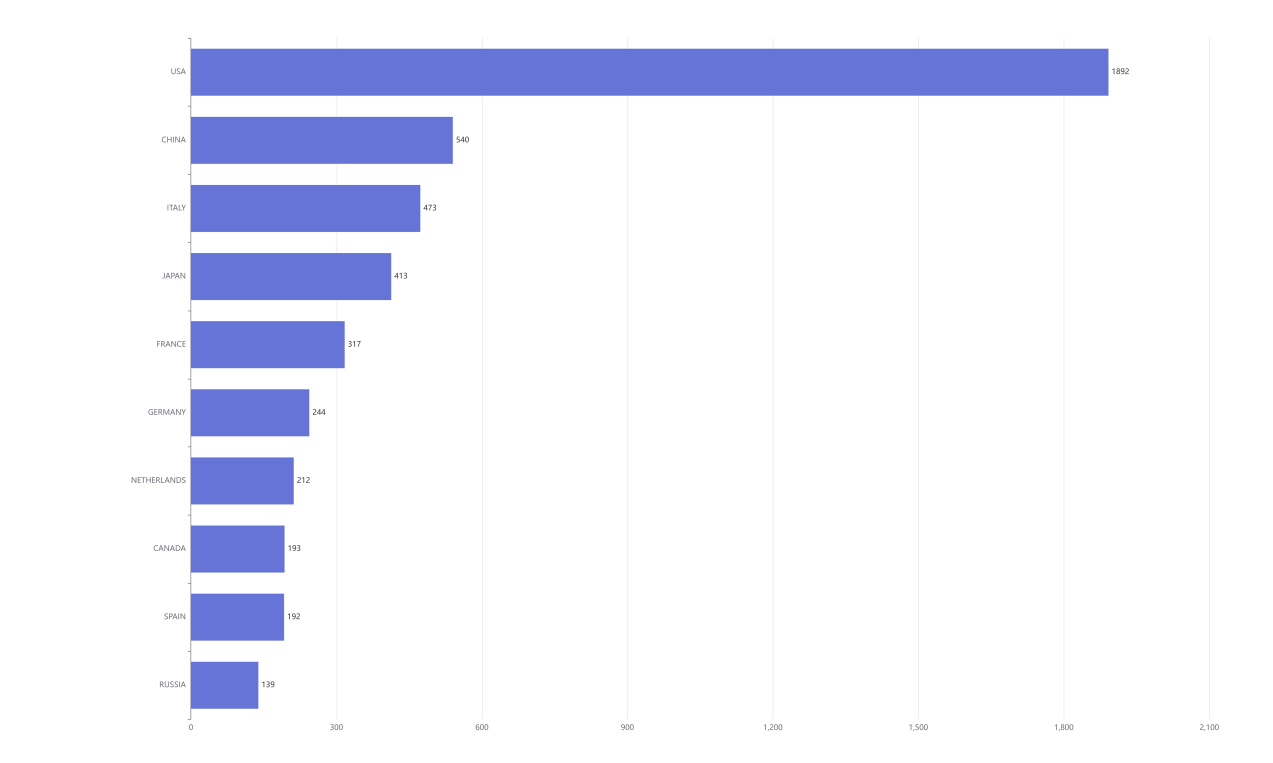
Fig. S7** Citation Rankings of Different Countries in the Disuse Muscle Atrophy from January 2010 to December 2024.

**
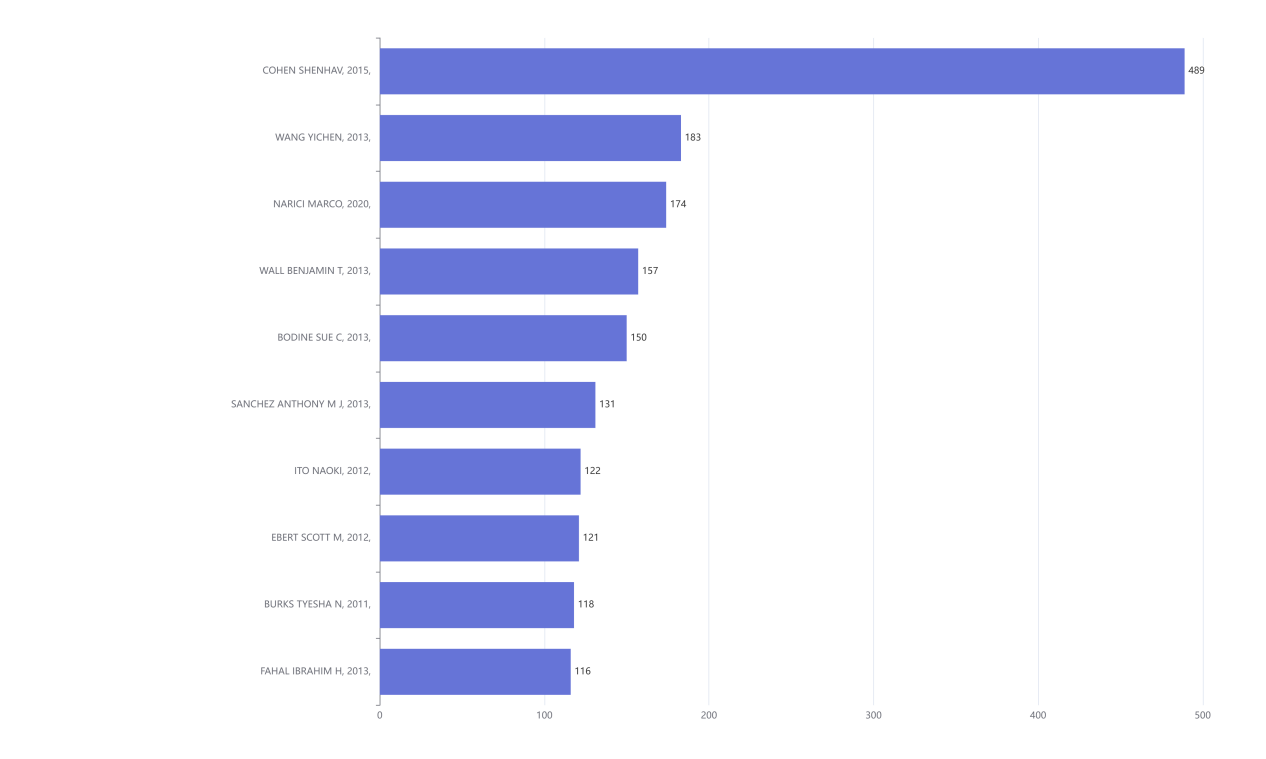
Fig. S8** Ranking of Cited Literature in the Disuse Muscle Atrophy from January 2010 to December 2024.


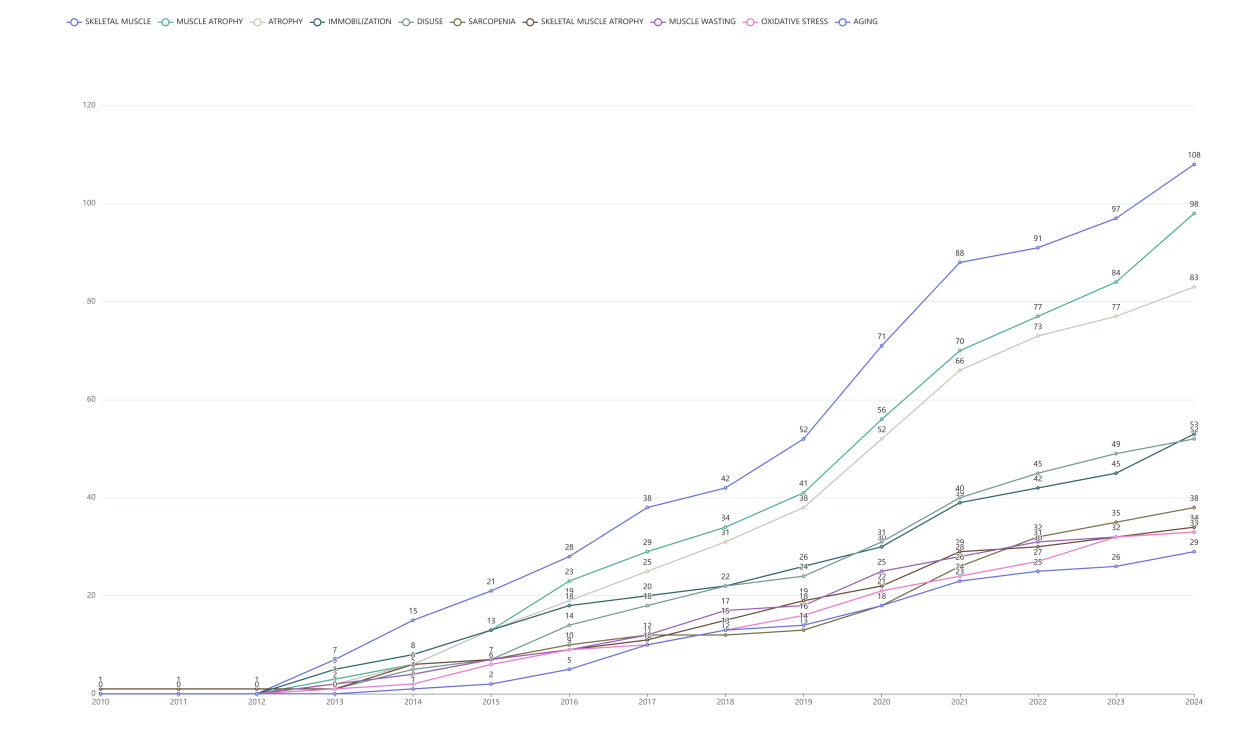
**Fig. S9** Frequency of Keywords in the Disuse Muscle Atrophy Literature Over Time: January 2010 to December 2024.

**
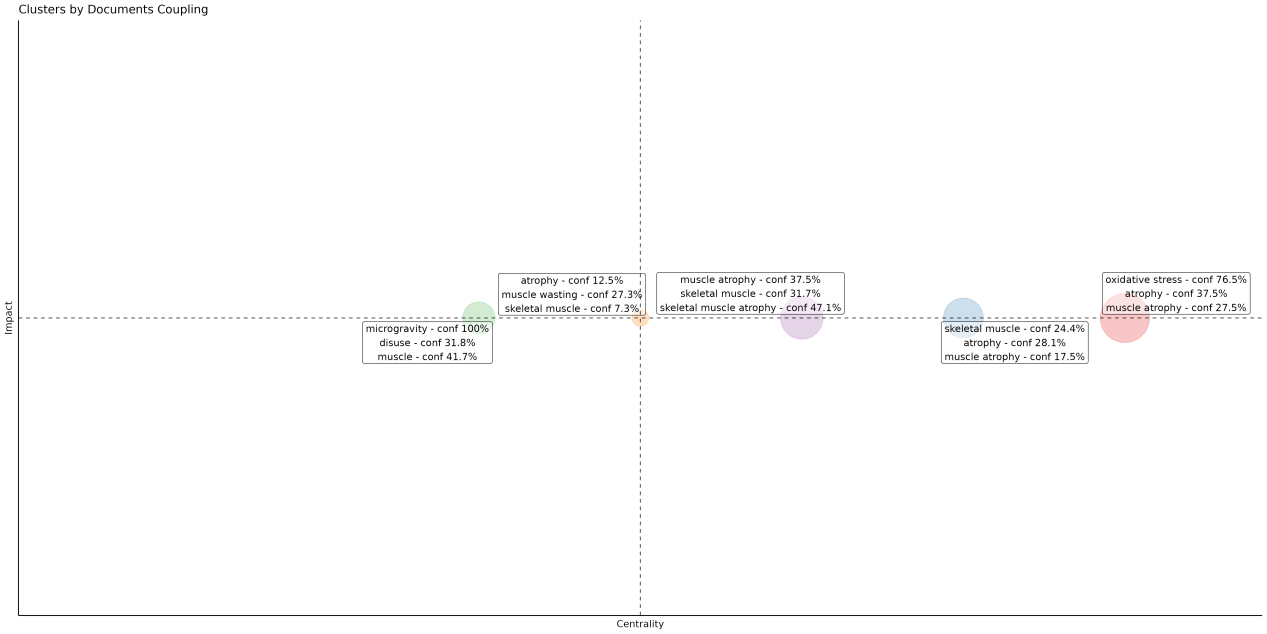
Fig. S10** Literature Coupling Clustering in the Disuse Muscle Atrophy from January 2010 to December 2024.

**
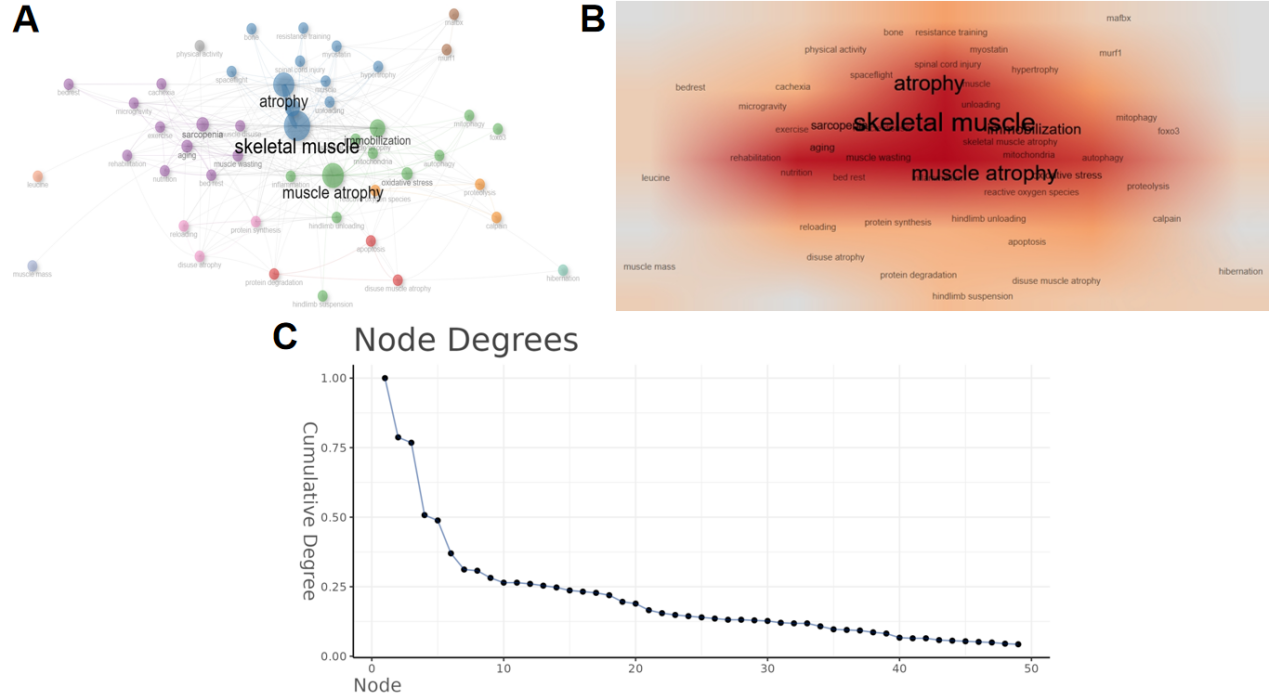
**

**Fig. S11** The co-occurrence network (A), density map (B), and degree distribution map (C) of keywords in the Disuse Muscle Atrophy from January 2010 to December 2024.

**Table S1** Key terms of Co-occurence Network

| **Node** | **Cluster** | **Betweenness** | **Closeness** | **PageRank** |
| --- | --- | --- | --- | --- |
| muscle atrophy | 3 | 260.3364 | 0.0169 | 0.0759 |
| skeletal muscle | 2 | 247.4598 | 0.0172 | 0.1037 |
| atrophy | 2 | 105.8153 | 0.0149 | 0.0764 |
| disuse | 2 | 59.8586 | 0.0133 | 0.0462 |
| sarcopenia | 4 | 50.9875 | 0.0132 | 0.0378 |
| immobilization | 3 | 46.1024 | 0.0135 | 0.0503 |
| oxidative stress | 3 | 21.4904 | 0.0125 | 0.0303 |
| exercise | 4 | 16.8278 | 0.0123 | 0.0234 |
| muscle wasting | 4 | 15.6567 | 0.0127 | 0.0226 |
| unloading | 2 | 13.863 | 0.0125 | 0.0271 |
| aging | 4 | 13.5887 | 0.0133 | 0.0332 |
| bed rest | 4 | 12.9156 | 0.0132 | 0.0247 |
| muscle | 2 | 12.0237 | 0.0123 | 0.0215 |

**
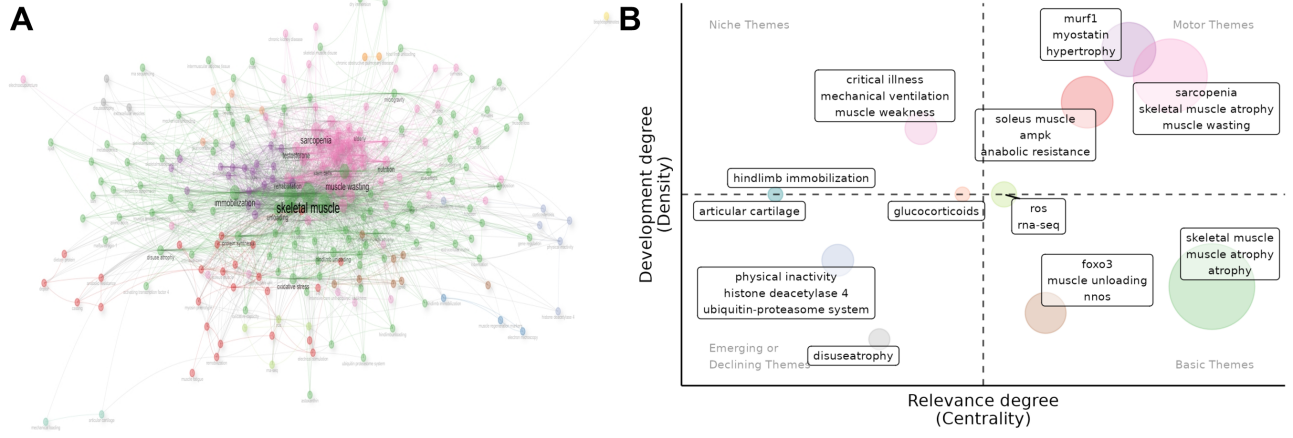
Fig. S12** Thematic Map of the Disuse Muscle Atrophy from January 2010 to December 2024.


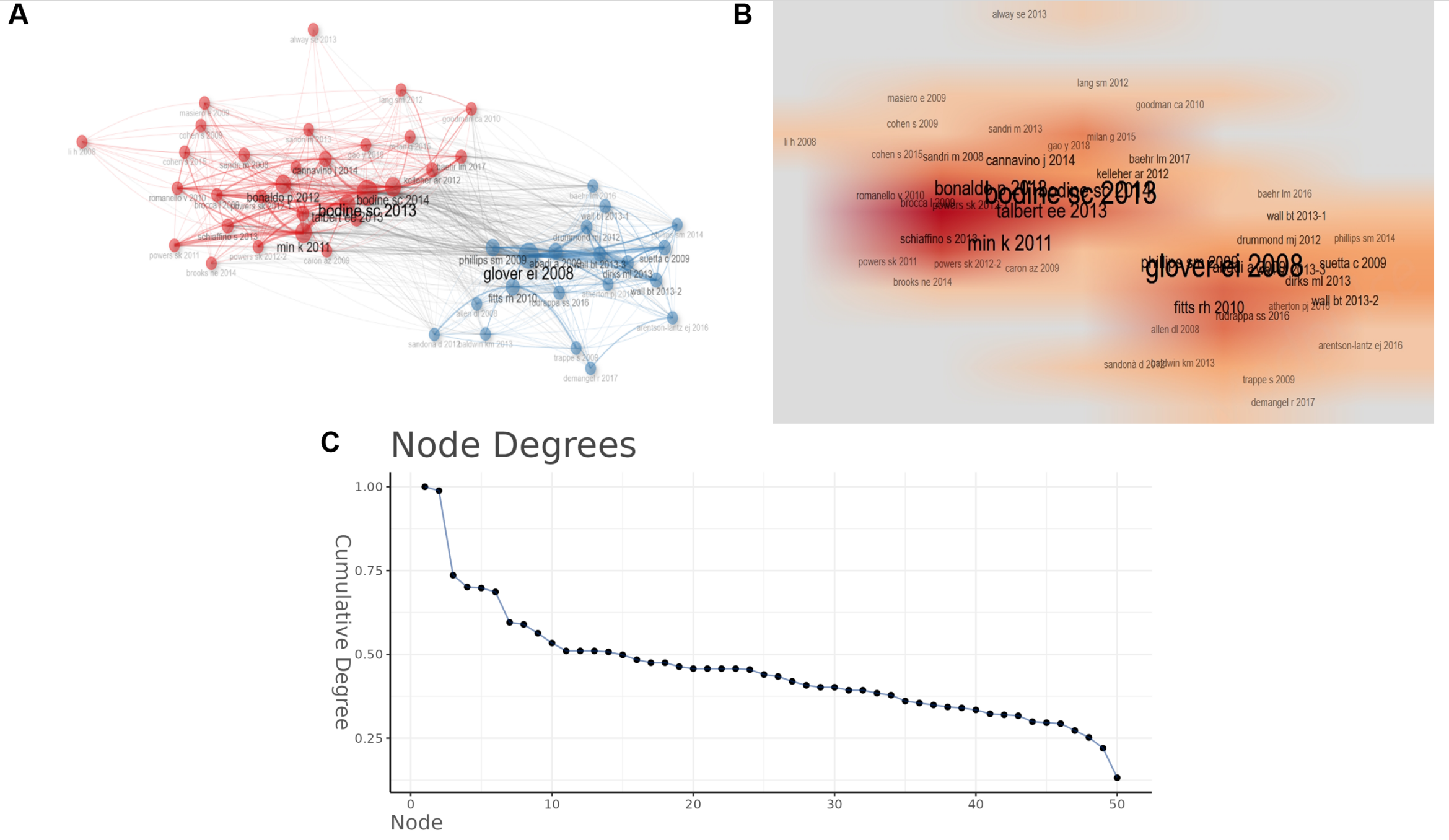
**Fig. S13** The co-citation network analysis in the Disuse Muscle Atrophy, spanning from January 2010 to December 2024, is constructed based on the frequency of simultaneous citations of literature.


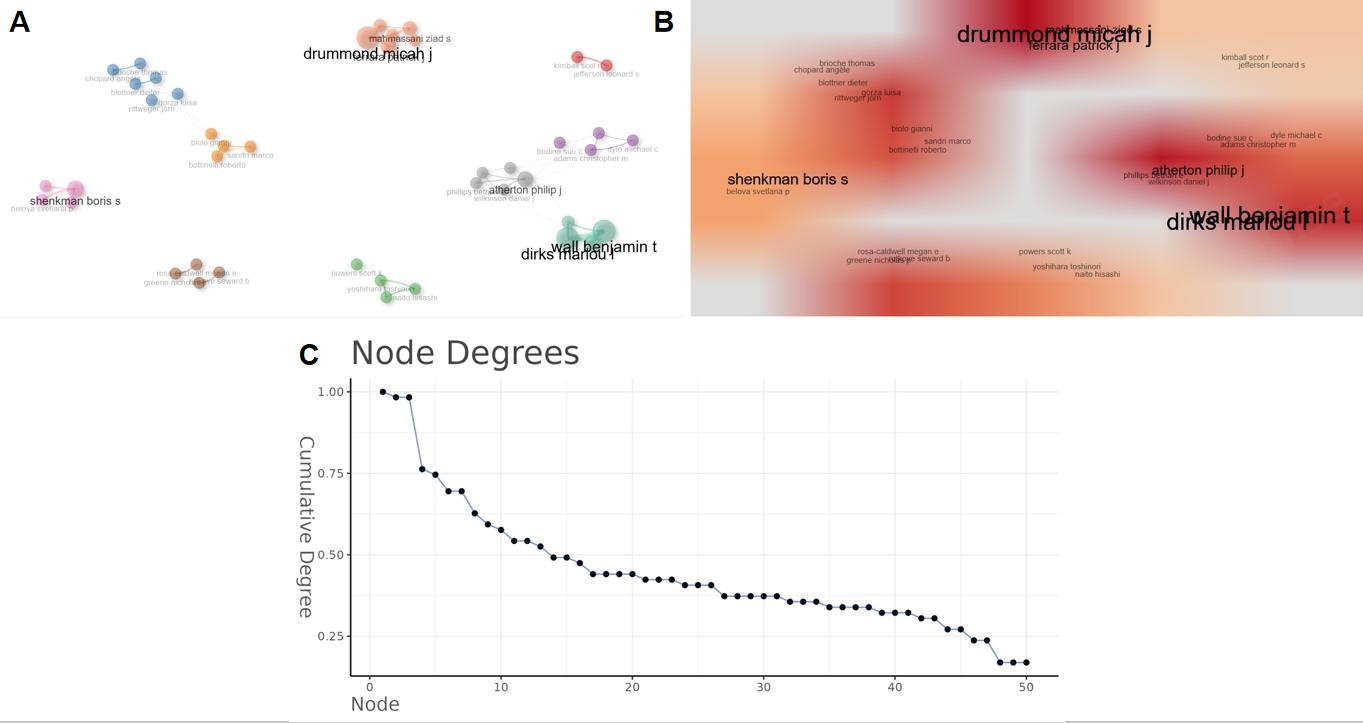
**Fig. S14** The collaborative network of literature in the Disuse Muscle Atrophy from January 2010 to December 2024 is analyzed based on joint research and publication activities among authors, institutions, or countries.


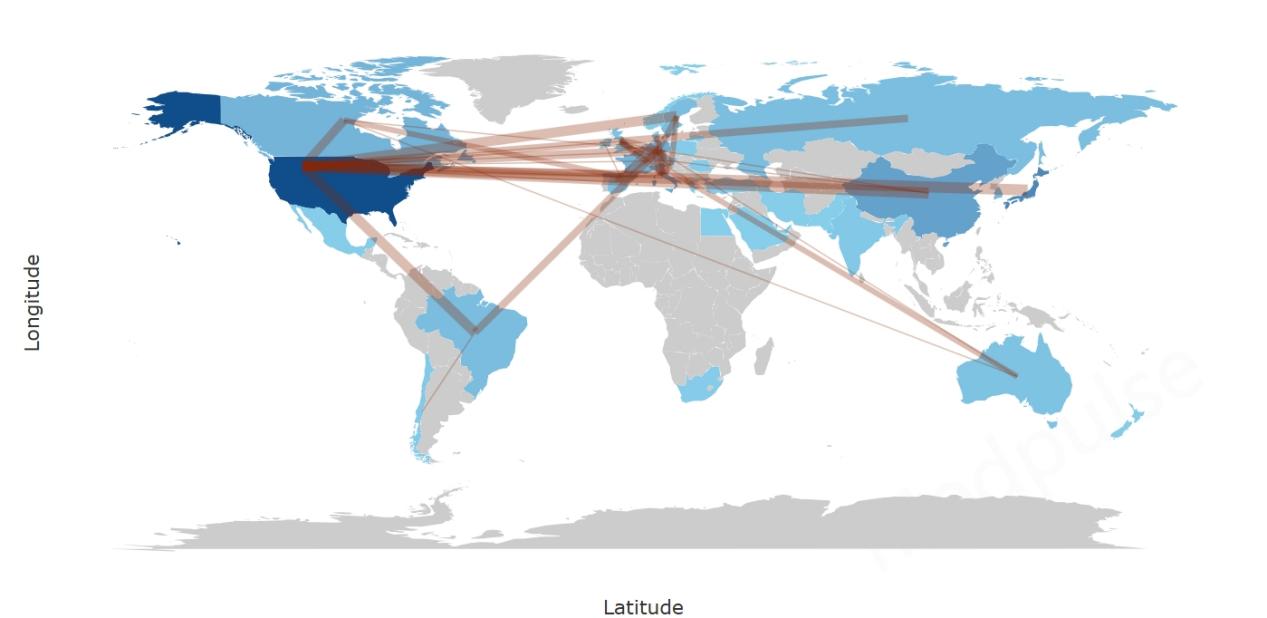


**Fig. S15** World Map of International Collaborations in the Disuse Muscle Atrophy from January 2010 to December 2024.

**Table S2** International Collaborations in the Disuse Muscle Atrophy from January 2010 to December 2024

| **From** | **To** | **Frequency** |
| --- | --- | --- |
| AUSTRALIA | DENMARK | 1 |
| AUSTRALIA | UNITED KINGDOM | 3 |
| BELGIUM | QATAR | 1 |
| BRAZIL | CHILE | 2 |
| BRAZIL | NEW ZEALAND | 1 |
| BRAZIL | SWEDEN | 1 |
| BRAZIL | SWITZERLAND | 1 |
| BRAZIL | UNITED KINGDOM | 1 |
| CANADA | AUSTRALIA | 2 |
| CANADA | AUSTRIA | 1 |
| CANADA | BRAZIL | 1 |
| CANADA | DENMARK | 1 |
| CANADA | IRAN | 1 |
| CANADA | NETHERLANDS | 1 |
| CANADA | POLAND | 1 |
| CANADA | SPAIN | 1 |
| CANADA | SWEDEN | 1 |
| CANADA | UNITED KINGDOM | 1 |
| CHILE | NEW ZEALAND | 1 |
| CHINA | AUSTRIA | 2 |
| CHINA | BRAZIL | 1 |
| CHINA | CANADA | 1 |
| CHINA | DENMARK | 1 |
| CHINA | GERMANY | 2 |
| CHINA | ITALY | 1 |
| CHINA | SWEDEN | 1 |
| DENMARK | AUSTRIA | 1 |
| FRANCE | AUSTRIA | 1 |
| FRANCE | BELGIUM | 1 |
| FRANCE | CANADA | 3 |
| FRANCE | DENMARK | 2 |
| FRANCE | GERMANY | 4 |
| FRANCE | IRELAND | 2 |
| FRANCE | NORWAY | 1 |
| FRANCE | QATAR | 1 |
| FRANCE | SPAIN | 4 |
| GERMANY | AUSTRALIA | 2 |
| GERMANY | AUSTRIA | 1 |
| GERMANY | BELGIUM | 1 |
| GERMANY | BRAZIL | 4 |
| GERMANY | CANADA | 2 |
| GERMANY | DENMARK | 2 |
| GERMANY | IRELAND | 1 |
| GERMANY | LITHUANIA | 1 |
| GERMANY | MEXICO | 1 |
| GERMANY | SLOVENIA | 4 |
| GERMANY | SOUTH AFRICA | 1 |
| GERMANY | SPAIN | 1 |
| GERMANY | SWEDEN | 3 |
| GERMANY | SWITZERLAND | 1 |
| GERMANY | UNITED KINGDOM | 5 |
| ITALY | AUSTRALIA | 1 |
| ITALY | AUSTRIA | 1 |
| ITALY | CANADA | 1 |
| ITALY | DENMARK | 3 |
| ITALY | FRANCE | 2 |
| ITALY | GERMANY | 9 |
| ITALY | LITHUANIA | 1 |
| ITALY | NETHERLANDS | 1 |
| ITALY | SLOVENIA | 3 |
| ITALY | SPAIN | 1 |
| ITALY | SWEDEN | 1 |
| ITALY | SWITZERLAND | 1 |
| ITALY | TURKEY | 1 |
| ITALY | UNITED KINGDOM | 3 |
| JAPAN | AUSTRIA | 1 |
| JAPAN | BRAZIL | 1 |
| JAPAN | CHINA | 1 |
| JAPAN | GERMANY | 1 |
| JAPAN | POLAND | 1 |
| JAPAN | SWEDEN | 1 |
| JAPAN | SWITZERLAND | 1 |
| KOREA | INDIA | 1 |
| NETHERLANDS | UNITED KINGDOM | 2 |
| PAKISTAN | SAUDI ARABIA | 1 |
| SPAIN | DENMARK | 1 |
| SWEDEN | AUSTRIA | 1 |
| SWEDEN | NORWAY | 1 |
| SWEDEN | SLOVENIA | 3 |
| SWEDEN | UNITED KINGDOM | 1 |
| TURKEY | UNITED ARAB EMIRATES | 1 |
| UNITED KINGDOM | CHILE | 1 |
| UNITED KINGDOM | LITHUANIA | 1 |
| UNITED KINGDOM | SLOVENIA | 2 |
| UNITED KINGDOM | SWITZERLAND | 1 |
| USA | AUSTRALIA | 1 |
| USA | AUSTRIA | 2 |
| USA | BELGIUM | 2 |
| USA | BRAZIL | 6 |
| USA | CANADA | 5 |
| USA | CHINA | 7 |
| USA | DENMARK | 1 |
| USA | FRANCE | 2 |
| USA | GERMANY | 5 |
| USA | IRAN | 1 |
| USA | IRELAND | 1 |
| USA | ISRAEL | 1 |
| USA | ITALY | 4 |
| USA | JAPAN | 7 |
| USA | KOREA | 1 |
| USA | RUSSIA | 4 |
| USA | SOUTH AFRICA | 1 |
| USA | SPAIN | 2 |
| USA | SWEDEN | 6 |
| USA | SWITZERLAND | 1 |
| USA | UNITED KINGDOM | 2 |
